# A systematic review of the influences of food store product placement on dietary-related outcomes

**DOI:** 10.1093/nutrit/nuaa024

**Published:** 2020-06-01

**Authors:** Sarah C Shaw, Georgia Ntani, Janis Baird, Christina A Vogel

**Affiliations:** 1 MRC Lifecourse Epidemiology Unit, Faculty of Medicine, University of Southampton, Southampton General Hospital, Southampton, UK; 2 NIHR Southampton Biomedical Research Centre, Faculty of Medicine, University of Southampton and University Hospital Southampton NHS Foundation Trust, Southampton, UK; 3 UK Arthritis Research UK/MRC Centre for Musculoskeletal Health and Work, Faculty of Medicine, University of Southampton, Southampton, UK

**Keywords:** diet, food stores, placement, sales, systematic review

## Abstract

**Context:**

Product placement strategies have been used to influence customers’ food purchases in food stores for some time; however, assessment of the evidence that these techniques can limit unhealthy, and promote healthy, food choices has not been completed.

**Objective:**

This systematic review aimed to determine how product placement strategies, availability, and positioning, in physical retail food stores located in high-income countries, influence dietary-related behaviors.

**Data Sources:**

From a search of 9 databases, 38 articles, 17 observational studies, and 22 intervention studies met the study inclusion criteria.

**Data Extraction:**

Two reviewers independently extracted data relating to study design, study population, exposures, outcomes, and key results. Each study was also assessed for risk of bias in relation to the research question.

**Data Analysis:**

Meta-analysis was not possible owing to heterogeneous study designs and outcomes. As recommended by Cochrane, results were synthesized in effect direction plots using a vote-counting technique which recorded the direction of effect and significance level according to the expected relationship for health improvement.

**Conclusions:**

The majority of studies showed that greater availability and more prominent positioning of healthy foods, or reduced availability and less prominent positioning of unhealthy foods, related to better dietary-related behaviors. A large number of results, however, were nonsignificant, which likely reflects the methodological difficulties inherent in this research field. Adequately powered intervention studies that test both the independent and additive effects of availability and positioning strategies are needed.

**Systematic Review Registration:**

PROSPERO registration no. 42016048826

## INTRODUCTION

The current food environment is obesogenic and encourages individuals to habitually overconsume foods in a way that is inconsistent with dietary recommendations.^1^ In the late 1990s, it was identified that modern food environments heavily promote the sale and intake of energy-dense, nutrient-poor foods and beverages.^2^ It took until 2007 for the first significant government document, the Foresight report, to highlight the key role of food environments in fueling obesity.^3^^,^^4^ Although published in the United Kingdom, this report has had international impact. Yet now, more than a decade later, food environments remain obesogenic and obesity levels continue to rise worldwide.^5^

Human behavior is often responsive to environmental stimuli in settings frequently visited.^6^ Food stores, such as supermarkets and corner stores, are the main sources of food for many people living in high-income countries; they are likely having a significant influence on the food choices of their consumers.^7^ Marketing strategies are used extensively in food stores and commonly comprise the 4 Ps of marketing: product, price, promotion, and placement.^8^ Product placement strategies have been used to influence customers’ purchases in these stores for some time, and their successful effects have been documented in the marketing literature.^9^^,^^10^ Assessment of the evidence that these techniques can be successfully used to limit unhealthy, and promote healthy, food choices has not yet been completed. Gray literature suggests that two-thirds of placement strategies are used to promote unhealthy food and beverages in supermarkets.^11^ Comprehensive assessment of academic research examining the health-related effects of placement strategies on store-level food sales, household-level food purchasing, or individual-level dietary outcomes would help guide future government intervention across the world. Some governments are already taking, or considering taking, legislative actions against food and beverage placement promotions. For example, Chapter 2 of the UK government’s Childhood Obesity Plan, released in 2018, included a population-level proposal to ban marketing strategies used in food outlets that promote the overconsumption of unhealthy foods and beverages.^12^

A number of systematic reviews have narratively summarized the influence of supermarket interventions on diet-related outcomes. Existing reviews have largely examined the evidence for intervention strategies related to product price and healthier product promotion, including product swaps, product signage, and product labeling.^13–18^ Only a very small number of studies included in these reviews assessed the role of product placement on dietary and food purchasing behaviors, and no quantitative evidence synthesis has been conducted to date. Reviews of observational research investigating the association between in-store food retail environments and dietary-related outcomes have not exhaustively examined product placement either – primarily because the literature in this area has grown rapidly since 2012, when 2 critical reviews on this topic were published.^19^^,^^20^ Policy makers would benefit from a systematic review of recent observational and intervention research investigating the role of product placement strategies in retail food stores on outcomes related to health, such as food sales, purchasing, dietary intake, or BMI.

According to the Typology of Interventions in Proximal Physical Micro-Environments (TIPPME), product placement consists of 2 distinct intervention types: availability and position.^21^ Availability describes the addition or removal of products to increase or decrease their variety, number, or range. Position refers to altering the position, proximity, or accessibility of products, rendering them easier or harder to engage with.

There is some evidence to indicate that public health strategies that alter environmental influences on health behaviors may be more equal in their effectiveness across socioeconomic groups than those requiring conscious or reflective engagement, which appear most beneficial for more advantaged groups.^22^ Assessing whether product placement strategies in retail food stores has a differential effect on dietary-related behaviors could provide important evidence to help address dietary inequalities. Thus, this systematic review aims to adopt a quantitative approach to answer the following questions:

1. Does an association exist between the availability of healthier and/or unhealthy food products in retail food stores and BMI, dietary behaviors, purchasing, and sales of these foods?
2. Does an association exist between the prominent positioning of healthier and/or unhealthy food products in retail food stores and BMI, dietary behaviors, purchasing, and sales of these foods?
3. Do these associations differ according to socioeconomic position?

## METHODS

Recommendations made by the Preferred Reporting Items for Systematic Reviews and Meta-Analyses (PRISMA) group were followed throughout this review.^23^Table S1 (please see the Supporting Information online) shows the PRISMA checklist for the review. This systematic review was registered with the Prospective Register for Systematic Reviews (PROSPERO) CRD: 42016048826.

### Data sources

Nine electronic databases were searched (Medline, DARE, Cochrane Database of Systematic Reviews, Cochrane Central Register of Controlled Trials, EBSCOhost, PsycINFO, Science Direct, EconLit, and Scopus). A combination of medical subject headings (MeSH) and free-text terms relating to “diet,” “feeding behavior,” “food,” “beverages,” “food supply,” and “food industry” were used to identify studies that described the association between in-store food environments and diet, sales, purchasing, and BMI outcomes in adults. All studies were published in English between January 2005 and February 2019. A landmark article describing different types of food environments in relation to diet and health was published in 2005, prior to this date little research was published in this area.^24^ The inclusion of this time frame captures the most recent literature in the research field, reflects current food store layouts and provides useful and applicable evidence for policy makers. The complete search strategy and list of search terms can be found in Table S2 (please see the Supporting Information online)*.* All titles and abstracts were screened by one author (S.C.S.) against the study PI(E)COS (population, intervention/exposure, comparison, outcome, and study design) criteria to ensure eligibility for inclusion (Table 1). Observational and intervention studies were included if they involved adult participants (aged 18 years and older), were conducted in high-income countries, included an exposure/intervention that investigated either the positioning or availability of food items in physical food stores and had an outcome relating to food sales, purchasing, dietary intake, or BMI. If it was unclear from the abstract alone whether an article was eligible for inclusion, the full text was reviewed. The bibliographies of the included studies were also screened for additional articles.

**Table 1:** PICOS criteria for inclusion and exclusion of studies

Parameter	Inclusion criteria	Exclusion criteria
General	Published in English Published between 2005 and February 2019	
Study design	Intervention studies Observation studies	Ecological studies
Population	Studies with individuals aged 18+ years as primary population High-income countries	Low-income countries
Exposure/ intervention	Positioning of food/ beverage items of foods which have clear association with health Availability of food/ beverage items	Studies that focus solely on price, food labelling and portion size Inadequate description of in-store placement measures
Setting	Supermarkets Convenience stores	Non-permanent location (outdoor/ farmers’ markets, pop-up stalls) Cafeterias Speciality food stores, eg, greengrocers, butchers
Outcomes	Dietary intake Food sales data Body composition	Food/ beverage outcomes (intake/sales) that do not clearly align with healthy eating guidelines

### Data extraction and risk-of-bias assessment

Data were extracted to capture the relevant information for the research questions. Separate data extraction forms were created for observational studies and intervention studies. The full text for each article was assessed independently by 2 reviewers (S.C.S. and C.A.V.). Details regarding the study characteristics (study design, setting, participant details, exposures, outcomes, results, and funding sources) were extracted.

Concurrent with the data extraction, risk of bias was assessed for each eligible article to determine the risk of bias in relation to the research questions. This process was conducted using predefined assessment criteria based on those described by the NHS Centre for Reviews and Dissemination.^25^ Separate risk-of-bias assessment criteria were used for observational and intervention studies. Thirteen domains in observational studies and 18 domains in intervention studies assessed the elements of study design, participant selection, attrition, assessment methodologies, statistical analyses, and handling of confounding effects (Tables S3 and S4; please see the Supporting Information online). A risk-of-bias score of +1 (low risk of bias), 0 (medium risk of bias), or −1 (high risk of bias) was allocated for each domain. If, for any reason, an element of the assessment criteria was not applicable, a score of 0 was applied for that domain. For example, a 0 rating was applied for the “cohort follow-up percentage” domain if the study was cross-sectional. The reviewers (S.C.S. and C.A.V.) compared the risk-of-bias assessment ratings for consistency. Any discrepancies were discussed in depth until a quality score was agreed. An overall risk-of-bias score was allocated to each study based on the number of “−1” ratings a study received. Intervention studies with 6 or more −1 ratings, and observational studies with 5 or more −1 ratings, were classed as having a high risk of bias overall. If the number of −1 scores was ≤2 for intervention studies or ≤1 for observational studies, the overall risk-of-bias score was classified as low. Intermediate ratings were allocated a moderate overall risk-of-bias score.

### Data synthesis

Separate summary tables were created for observational and intervention studies (Tables S5 and S6; please see the Supporting Information online). Each study was categorized according to the placement strategy (availability or positioning) of the exposure or intervention. Availability and positioning were defined according to the TIPPME recommendations.^21^

Studies were further classified to reflect whether the exposure/intervention focused on the “placement of healthy foods” or “placement of unhealthy foods.” These categorizations were based on the Eatwell Guide (Public Health England).^26^ Foods that were inadequately described or did not clearly align with this guide were excluded from this review. For example, popcorn, vegetarian pepperoni, and wheat-square cereal – covered in the study by Holmes et al^27^ – were not included in the quantitative assessment as an expected direction for health outcomes could not be determined. Exposures/interventions that considered both healthy and unhealthy food items together were categorized under “placement of healthy and unhealthy food.”

Meta-analysis was not possible owing to the heterogeneity of the study designs, exposures/interventions, and outcomes. A vote-counting method was therefore used to summarize the findings of this review. Cochrane’s advice for accurate vote counting was followed throughout this review and requires that each study’s effect estimates are categorized according to their direction in terms of showing a health benefit or harm, in order to produce a standardized binary metric.^28^ This systematic review hypothesized that greater availability and/or more prominent positioning of healthy foods resulted in a benefit for health through greater sales/purchasing/consumption of the healthy food, reduced sales/purchasing/consumption of unhealthy food items, or lower BMI. In addition, this review also hypothesized that reduced availability and/or no prominent positioning of unhealthy foods resulted in a benefit for health through decreased sales/purchasing/consumption of the unhealthy foods, increased sales/purchasing/consumption of healthy foods, or lower BMI. Each outcome result from an article was classified as either positive (supports hypothesis) or negative (rejects hypothesis). In cases where the direction of the outcome result could not be determined, results were categorized as inconclusive. Each article’s results were further classified according to the significance level (significant *P* ≤ 0.05 or nonsignificant *P* > 0.05). Only results that were deemed to be relevant to the research question were extracted during the data synthesis process. The vote-counting results were summarized visually using bar charts and in detail using effect direction plots, as recommended by Cochrane.^28^ Effect direction plots indicate studies that report on more than one similar outcome (diet, sales, purchasing, BMI) in a way that is not captured by a bar chart. Arrows were used in effect direction plots to represent the combined direction and significance level of outcomes for each study. The method of combining results was based on previous criteria for variation in effect and significance^29^:

If ≥70% of outcomes report similar direction use an arrow (▴ [positive] or ▾ [negative]) to represent the direction.

- If <70% of outcomes report a similar direction, use a diamond (⋄) to represent inconsistent results.
- If effect direction similar AND >60% outcomes are statistically significant, use a solid arrow (▴) to represent a significant result.
- If effect direction similar AND <60% of outcomes are statistically significant, use a hollow arrow (▵) to represent a nonsignificant result.

## RESULTS

### Search results

**Figure 1:**
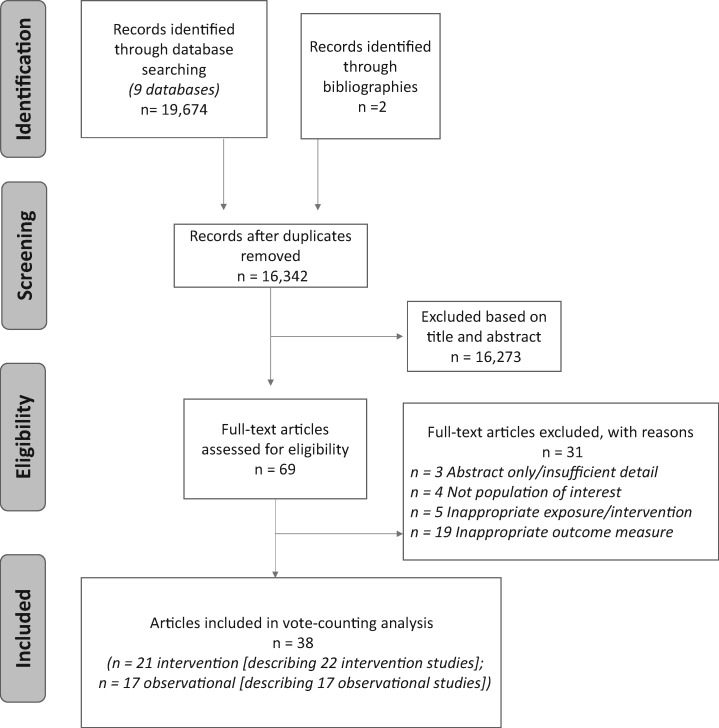
Flow diagram of the literature search process

Figure 1 is a PRISMA diagram representing the literature search process. After removal of duplicates, 16 342 references were identified from the 9 databases searched. A further 2 articles were included from bibliographic review. All titles and abstracts were screened and 69 full-text articles were reviewed for eligibility. Thirty-one articles were excluded because of insufficient detail or inappropriate population, exposure/intervention, or outcome. Overall, 38 articles were deemed appropriate for inclusion. These articles described 17 observational studies and 22 intervention studies. Two of the intervention studies were reported in the same article but used different data sources and addressed different research questions. This article was therefore treated as 2 separate studies in this review (these studies are presented throughout as 'Ejilerskov et al 2018a1' and 'Ejilerskov et al 2018a2').^30^

### Study characteristics

#### Observational studies.

Publication dates of the included observational studies ranged from 2008 to 2017. In total, 13 769 participants and over 1487 food stores were studied in the included observational literature. All but one of the observational studies had a cross-sectional design (n = 16; 94%). Table S5 (please see the Supporting Information online) provides a detailed summary of the study design, study setting, participant demographics, key findings, and quality for all observational studies.

#### Intervention studies.

The intervention studies were published between 2009 and 2019. In total, over 40 571 participants and 289 food stores were included. The study designs varied greatly between the intervention studies: 4 studies (18%) described randomized control trials,^31–34^ 4 (18%) a quasi-experimental design,^35–38^ 7 (32%) a repeated cross-sectional design,^30^^,^^39–45^ and 4 (18%) alternating treatment designs,^46–49^ while 2 (9%) used time-series analyses^27^^,^^30^ and 1 (5%) was a natural experiment.^30^

Table S6 (please see the Supporting Information online) provides a detailed summary of the study design, study setting, participant demographics, key findings, and quality for all intervention studies.

### Exposures/interventions and outcomes

#### Observational studies.

Of the 17 observational studies, 9 (53%) focused on supermarkets,^50–58^ 6 (35%) on convenience stores,^59–64^ and 2 (12%) on both supermarkets and convenience stores.^65^^,^^66^ Fourteen observational articles (82%) assessed availability.^50^^,^^52–54^^,^^57–66^ While heterogeneous measures were used, all observational studies conducted in-store audits to assess food placement strategies. Five studies (29%) assessed availability by measuring the shelf space dedicated to specific food items.^50^^,^^59^^,^^60^^,^^64^^,^^66^ Length of shelf space (m) was the most common measure of shelf space, but total shelf space (length × depth, m^2^) was also used in 2 studies (n = 2/5; 40%).^50^^,^^64^ Eight observational studies (47%) used cumulative scoring techniques to assess in-store availability.^52–54^^,^^60–62^^,^^64^^,^^65^ Five studies (n = 5/8; 75%) used modified versions of the Nutrition Environment Measures Survey in Stores (NEMS-S).^52–54^^,^^62^^,^^65^ NEMS-S assesses the availability, price, and quality of healthy food items within stores. The modifications varied greatly between studies, with each study assessing different items; none reported validity testing on these modified NEMS-S tools.The “healthy food supply” score was used in 2 studies (n = 2/8; 25%).^60^^,^^61^ This score is similar in structure to the NEMS-S tool, assessing availability, variety, price, and quality, but focuses on subsidized items approved for the US Women, Infants, and Children program. Six studies (n = 6/14; 43%) assessed product variety as a measure of availability, ^50^^,^^57^^,^^59^^,^^60^^,^^62^^,^^63^ 5 studies (n = 5/6; 83%) tallied the number of different varieties of fruit and vegetables available,^50^^,^^59^^,^^60^^,^^62^^,^^63^ and 1 study (n = 1/6; 17%) assessed the number of different varieties of chocolate and confectionery available.^57^

Five observational studies (29%) examined food positioning strategies.^51^^,^^55^^,^^56^^,^^60^^,^^63^ Of these, all 5 (100%) measured store positioning – namely, checkout areas (n = 3/5; 60%),^51^^,^^55^^,^^60^ store entrances (n = 2/5; 40%),^60^^,^^63^ special floor displays (n = 1/5; 20%),^51^ and end-of-aisle displays (n = 3/5; 60%).^51^^,^^55^^,^^56^ One of these studies (n = 1/5; 20%) additionally measured shelf positioning by assessing whether bottled water was placed at eye level.^63^ One study described the development, reliability, and validity of the GroPromo tool.^55^ This tool assesses the presence of food items in 9 locations within food stores which vary in their level of prominence. It was the only validated tool identified in this review to assess positioning. Four other studies (n = 4/5; 80%) used dichotomized variables (Yes/No) to record whether specific types of food items were positioned in prominent store or shelf locations.^51^^,^^56^^,^^60^^,^^63^

Sales-related outcome measures were used in 6 observational studies (35%),^55–57^^,^^60^^,^^62–64^ 8 studies (47%) assessed dietary-related outcomes,^50^^,^^52^^,^^53^^,^^58^^,^^59^^,^^61^^,^^65^ and 4 studies (24%) assessed BMI.^51^^,^^53^^,^^54^^,^^66^ For those evaluating sales and purchasing, objective store-level sales data was the outcome in only one study (n = 1/7; 14%).^56^ The remainder (n = 6/7; 86%) recorded individual-level purchases via store exit interviews and shopping bag audits. Self-reported dietary data were collected using a number of different dietary tools. The majority of studies (n = 6/7; 86%) that examined dietary data used fruit and vegetable measures as the primary outcome.^50^^,^^52^^,^^53^^,^^58^^,^^59^^,^^61^ Other dietary measures included sugar-sweetened beverages (n = 3/7; 43%),^51^^,^^52^^,^^61^ chocolate and confectionery (n = 1/7; 14%),^58^ and biscuits and cakes (n = 1/7; 14%).^52^ One study (n = 1/7; 14%) reported a 120-item food frequency questionnaire used to produce 2 dietary pattern scores: one score described a high-quality diet (high intakes of whole grains and fruits) and the other described a low-quality diet (high intakes of high-fat foods and processed meats).^65^ One study (n = 1/7; 14%) used a novel measure – reflection spectroscopy – to objectively assess skin carotenoids as a marker of fruit and vegetable consumption in addition to self-reported fruit and vegetable consumption.^61^ Four studies considered BMI as an outcome.^51^^,^^53^^,^^54^^,^^66^

#### Intervention studies.

In accordance with this review’s inclusion criteria, all interventions were conducted in physical food retail stores; 13 articles (n = 13/22; 59%) reported interventions taking place in supermarkets,^27^^,^^30^^,^^32^^,^^35^^,^^38^^,^^39^^,^^42^^,^^45–49^ 8 (n = 8/22; 36%) in convenience stores,^31^^,^^34^^,^^37^^,^^40^^,^^41^^,^^43^^,^^44^ and 1 (n = 1/22; 5%) in both supermarkets and convenience stores.^36^ Overall, the 22 intervention studies involved 243 intervention stores and 43 control stores. Eight studies (n = 8/22; 36%) did not include a control group.^27^^,^^41^^,^^44–49^ None of the intervention studies mentioned sample size calculations. Of the 14 studies (n = 14/22; 64%) that included a comparator group, 1 study (n = 1/14; 7%) had one control checkout per store to act as a comparison,^39^ another study (n = 1/14; 7%) included delayed treatment controls,^31^ 4 (n = 4/14; 29%) used unmatched control stores,^34^^,^^36–38^ and 8 (n = 8/14; 57%) used matched control stores based on store characteristics, geographic location, and food product sales.^30^^,^^32^^,^^33^^,^^35^^,^^40^^,^^42^^,^^43^ Nine intervention studies (n = 9/22; 41%) incorporated availability in the treatment condition,^31^^,^^32^^,^^36–38^^,^^41^^,^^43^^,^^44^^,^^49^ and 18 (n = 18/22; 82%) included positioning components.^27^^,^^30–35^^,^^38–42^^,^^45–49^ Thirteen studies (n = 13/22; 59%) were not solely placement interventions and contained additional intervention features such as social marketing campaigns, staff training, shelf labeling, food demonstrations, signage, and financial incentives.^27^^,^^31–34^^,^^36–38^^,^^40^^,^^41^^,^^44^^,^^47^

The majority of intervention studies (n = 7/9; 78%) examining availability focused on increasing the availability of healthy foods.^31^^,^^36^^,^^37^^,^^41^^,^^43^^,^^44^ One study (n = 1/9; 11%) increased the availability of crisps,^49^ and 2 studies (n = 2/9; 22%) manipulated the availability of both healthy and unhealthy items.^32^^,^^38^ Of the 18 studies that focused on positioning, 4 (n = 4/18; 22%) manipulated shelf positioning, particularly the role of positioning food at eye level.^32^^,^^35^^,^^46^^,^^49^ The majority (n = 13/18; 72%), however, focused on product position within the store.^27^^,^^30^^,^^31^^,^^33^^,^^38–40^^,^^42^^,^^45^^,^^47–49^ The most common store position tested was the checkout, investigated in 7 studies (n = 7/13; 54%)^30^^,^^38^^,^^39^^,^^42^^,^^45^^,^^47^^,^^48^; 3 studies (n = 3/13; 23%) examined front-of-store positioning,^33^^,^^38^^,^^40^ and 4 (n = 4/13; 29%) investigated island displays.^27^^,^^31^^,^^38^^,^^49^ One study assessed both shelf and store positioning.^34^

The majority of intervention studies (n = 20/22; 91%) used sales-related outcomes.^27^^,^^30^^,^^32–35^^,^^37–49^ Only 4 studies (n = 4/22; 18%) measured diet-related outcomes,^31^^,^^36^^,^^40^^,^^43^ and 1 (n = 1/22; 5%) assessed BMI.^43^ Most studies (n = 15/20; 75%) that used sales-related outcomes collected data at the store level. Of these, 9 (n = 9/15; 60%) used objective store sales data,^27^^,^^32^^,^^35^^,^^38^^,^^45–49^ 3 studies (n = 3/15; 20%) conducted bag checks and checkout observations,^39^^,^^43^^,^^44^ 2 studies (n = 2/15; 13%) relied on store manager reported sales,^34^^,^^37^ and 1 study (n = 1/15; 7%) used store sales provided by the state department relating to women, infants, and children.^33^ Self-reported household-level purchasing data from Kantar Worldpanel were applied in 3 studies (n = 3/20; 15%).^30^^,^^42^ Another 3 studies (n = 3/20; 15%) relied on self-reported purchases of food items.^33^^,^^40^^,^^41^

Of the 4 studies that assessed dietary outcomes, 1 (n = 1/4; 25%) used a “healthy food getting” variable that assessed self-reported consumption of 26 healthy foods over the past 30 days.^36^ The 3 remaining studies (n = 3/4; 75%) assessed self-reported fruit and vegetable consumption,^31^^,^^40^^,^^43^ with 1 also including self-reported sugar-sweetened beverage consumption.^43^ A validated questionnaire was used in only one study^31^; however, another used reflection spectroscopy to objectively assess skin carotenoids as a marker of fruit and vegetable consumption.^43^

### Key findings

**Figure 2:**
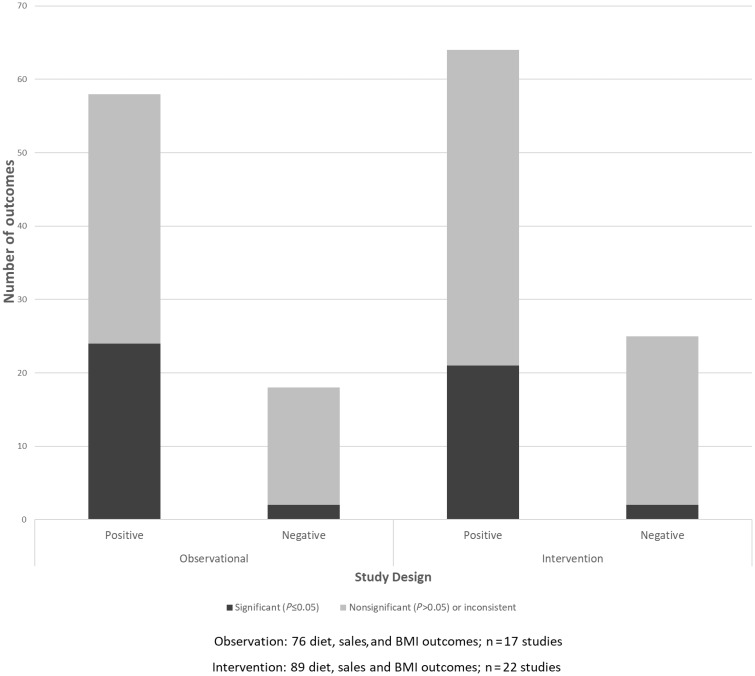
Summary of placement strategy vote-counting results from observational and intervention studies in relation to the review hypothesis

Figure 2 visually presents the quantitative vote-counting results, incorporating 76 diet, sales, and BMI outcomes from 17 observational studies, and 89 outcomes from 22 intervention studies. More than three-quarters of the observational outcomes (76%) showed positive findings supporting the review hypotheses; approximately one-quarter (24%) showed negative findings that did not support the review hypotheses.Of all observational findings, 66% were nonsignificant (59% positive nonsignificant outcomes, 89% negative nonsignificant outcomes). Almost three-quarters of the intervention outcomes (72%) showed positive findings supporting the review hypotheses; approximately one-quarter of the intervention outcomes (28%) showed negative findings that did not support the study hypotheses. A large proportion of the intervention outcomes (74%), however, were nonsignificant (67% positive nonsignificant outcomes, 92% negative nonsignificant outcomes).

### Research question 1: Does an association exist between the availability of healthier and/or unhealthy food products in retail food stores and BMI, dietary behaviors, purchasing, and sales of these foods?

#### Observational studies.

As shown by the effect direction plot in Table 2, 14 observational studies (82%) assessed food availability,^50–55^^,^^57–66^ of which over half (n = 8/14; 57%)^50^^,^^52^^,^^59–64^ found that product availability in retail food outlets was associated with outcomes that supported the review hypotheses and showed health benefits (3 positive significant [+s] outcomes, 5 positive nonsignificant [+ns] outcomes). Two studies reported results that did not support the hypotheses (2 negative nonsignificant [−ns]), and 4 studies reported inconsistent results.

**Table 2:** Effect direction plot of observational studies

Author, year	Study design	Socioeconomic status	Sample size	Placement of healthy foods	Placement of unhealthy foods	Placement of healthy and unhealthy foods	Outcome type^b^	Effect direction and significance^c^	Risk of bias
Bodor et al (2008)^59^	CS	Low	102	A			Diet	▵^6^	Low
Caldwell et al (2009)^50^	LT	Not provided	130	A			Diet	▵_6_	High
Caspi et al (2017)^60^	CS	Not provided	594	A P		A	Sales Sales Sales	▵_7_ ▵_2_ ▵_2_	Low
Cohen et al (2015)^51^	CS	Low	980	P	P		BMI BMI	▵_2_ ▿	Moderate
Franco et al (2009)^65^	CS	Varied	759	A			Diet	⋄^6^	Low
Gustafson et al (2011)^53^	CS	Not provided	186	A A			BMI Diet	▿ ▿	Low
Gustafson et al (2013)^52^	CS	Low	121	A			Diet	▵_4_	Low
Jani et al (2018)^54^	CS	Not provided	3817	A			BMI	⋄_2_	High
Jilcott Pitts et al (2017^)61^	CS	Not provided	479	A			Diet	▵_4_	Low
Kerr et al (2012)^55^	CS	Varied	637		P		Sales	▴_2_	Moderate
Martin et al (2012)^62^	CS	Low	372	A			Sales	▴_2_	Low
Nakamura et al (2014)^56^	CS	Not provided	1^a^	P	P		Sales Sales	▴_2_ ▴	Moderate
Rose et al (2009)^66^	CS	Not provided	1243	A	A		BMI BMI	⋄_3_ ▵_3_	Low
Ruff et al (2016)^63^	CS	Varied	1904	P A			Sales Sales	▿_4_ ▴_5_	Low
Sanchez-Flack et al (2017)^64^	CS	Low	369	A			Sales	▴_4_	Moderate
Thornton et al (2010)^58^	CS	Varied	1082	A			Diet	▿_4_	Moderate
Thornton et al (2011)^57^	CS	Varied	1007		A		Diet	⋄_2_	Moderate

^a^ No. of stores rather than no. of participants.

^b^ Sales represents sales/purchasing.

^c^ ▴ Positive result (*P*  < 0.05); ▵ positive result (*P*  > 0.05); ▾ negative result (*P*  < 0.05); ▿ negative result (*P*  > 0.05); ⋄ inconsistent results.

Number of outcomes within each category is 1 unless indicated in subscript beside effect direction.

Reported effect direction and significance for multiple outcomes: - All outcomes report effect in same direction and with same level of statistical significance **OR** - Where direction of effect varies across multiple outcomes:  Overall result direction determined if ≥70% of outcomes report similar direction; Overall result significance level determined if ≥70% of outcomes report similar statistical significance  Inconsistent findings rated as inconsistent if <70% of outcomes report consistent direction of effect (⋄).

Of the 13 studies (n = 13/14; 93%) that assessed availability of healthy food products,^50^^,^^52–54^^,^^58–66^ 57% (n = 8/14) showed results in the expected direction for health (5 +ns, 3 +s).^50^^,^^52^^,^^59–64^ In addition, one study (n = 1/14; 7%) assessed the availability of healthy food items separately to unhealthy items. The results showed a nonsignificant positive relationship between unhealthy food availability and BMI but inconsistent findings for the availability of healthy foods and BMI.^66^ One study showed that having a greater proportion of shelf space dedicated to fruit and vegetables, compared to unhealthy drinks and snacks, was associated with healthier purchases (+ns).^60^ One study assessed the availability of chocolate and confectionery, finding an overall inconsistent relationship with the consumption of these items – specifically, a nonsignificant positive association for confectionery exposure and confectionery consumption but no clear trend between chocolate exposure and chocolate consumption.^57^

The 4 studies that considered purchasing outcomes demonstrated the most consistent support of the review hypotheses; 80% (n = 3/4) found significant positive associations^62–64^ and 20% (n = 1/4) nonsignificant positive associations.^60^ Half of the studies (n = 4/8; 50%) with diet outcomes indicated a relationship with food availability in the expected direction for health benefit; however, none were statistically significant.^50^^,^^52^^,^^59^^,^^61^ BMI showed no clear relationship with food availability; 2 of the 3 studies showed inconsistent results,^54^^,^^66^ and one identified a nonsignificant relationship between greater healthy food availability and higher BMI.^53^

#### Intervention studies.

Table 3 shows the effect direction plot for intervention studies. Four intervention studies (n = 4/22; 18%) described manipulation of food availability.^36^^,^^37^^,^^43^^,^^44^ Of these, none showed results in the expected direction for health benefit. One study had results in the unexpected direction (negative significant, −ns),^36^ and 3 showed inconsistent results.^37^^,^^44^^,^^61^ All 4 of these studies targeted the availability of healthy foods, with none reducing the availability of unhealthy food items; additionally, all 4 were implemented as part of multicomponent interventions.^36^^,^^37^^,^^43^^,^^44^

**Table 3:** Effect direction plot of intervention studies

Author, year	Study design	SES status	Sample Size	Placement of healthy foods	Placement of unhealthy foods	Placement of healthy and unhealthy	Outcome type^c^	Effect direction and significance^d^	Risk of Bias
Adam et al (2017)^35^	QE	Not provided	10^a^	P	P		Sales Sales	▴ ▵	High
Adjoian et al (2017)^39^	RCS	Low	3^a^	P			Sales	▴^2^	High
Albert et al (2017)^40^	RCS	Low	550			P_M_	Sales Diet	▵^3^ ▵	High
Ayala et al (2013)^31^	RCT	Low	119	AP_M_			Diet	▵^3^	High
Dannefer et al (2012)^41^	RCS	Low	294	AP_M_			Sales	▵^2^	High
De Wijk et al (2016)^46^	AT	Not provided	2^a^			P	Sales	⋄	High
Ejlerskov et al (2018a1)^30^	TS	Varied	30,000^b^			P	Sales	▴^2^	High
Ejlerskov et al (2018a2)^30^	NE	Varied	30,000^b^			P	Sales	▴	High
Ejlerskov et al (2018)^42^	RCS	Varied	30,000^b^			P	Sales	⋄	High
Foster et al (2014)^32^	RCT	Low	8^a^		AP_M_		Sales	▵^13^	Moderate
Gittelsohn et al (2010)^36^	QE	Low	83	A_M_			Diet	▿	High
Holmes et al (2012)^27^	TS	Not provided	1^a^	AP_M_			Sales	⋄^16^	High
Jilcott Pitts et al (2018)^43^	RCS	Low	223	A_M_ A_M_ A_M_			Sales Diet BMI	▿ ⋄^3^ ▿	High
Lawman et al (2015)^44^	RCS	Low	8671	A_M_			Sales	⋄^3^	High
Sigurdsson et al (2009)^49^	AT	Not provided	2^a^		AP		Sales	▵^2^	High
Sigurdsson et al (2011)^47^	AT	Not provided	2^a^	P		P P_M_	Sales Sales Sales	▵ ⋄ ▵	High
Sigurdsson et al (2014)^48^	AT	Not provided	2^a^	P		P_M_	Sales Sales	▵ ▵	High
Song et al (2009)^37^	QE	Low	13^a^	A_M_			Sales	⋄^10^	High
Thorndike et al (2017)^33^	RCT	Low	575			P_M_	Sales	▵_2_	Moderate
Toft et al (2017)^38^	QE	Not provided	3^a^			AP_M_	Sales	▵_6_	High
Wensel et al (2018)^34^	RCT	Low	10^a^	P			Sales	▿^2^	High
Winkler et al (2016)^45^	RCS	Low	4^a^			P	Sales	⋄^5^	High

^a^ Related to no. of stores.

^b^ Estimated sample size.

^c^ Sales represents sales/purchasing.

^d^ ▴ Positive result (*P*  < 0.05); ▵ positive result (*P*  > 0.05); ▾ negative result (*P*  < 0.05)**;** ▿ negative result (*P*  > 0.05); ⋄ inconsistent results.

Number of outcomes within each category is 1 unless indicated in subscript beside effect direction.

Reported effect direction and significance for multiple outcomes: - All outcomes report effect in same direction and with same level of statistical significance **OR** - Where direction of effect varies across multiple outcomes:  Overall result direction determined if ≥70% of outcomes report similar direction; Overall significant level determined if ≥70% of outcomes report similarstatistical significance   Inconsistant findings rated as inconsistent if <70% of outcomes report consistent direction of effect (⋄).

*Abbreviations:* A, availability; AP, availability and positioning; AT, alternating treatment; M, multicomponent study; NE, natural experiment; P, positioning; QE, quasi-experimental; RCS, repeated cross-sectional; RCT, randomized controlled trial; TS, time series.

For the studies assessing sales/purchasing (n = 3/4, 75%), inconsistent results were observed in 2 studies,^37^^,^^44^ and one study showed that increasing the availability of healthy food items resulted in reduced sales of these items (−ns).^43^ In the 2 studies assessing dietary outcomes (n = 2/4; 50%), one found results in the unexpected direction (−ns),^36^ and the other showed inconsistent results.^43^ The one study (n = 1/4; 25%) that assessed BMI found that improving the availability of fruit, vegetables, low-fat milk, and whole-grain products in convenience stores resulted in a nonsignificant increase in BMI among intervention customers compared with control customers.^43^

### Research question 2: Does an association exist between the prominent positioning of healthier and/or unhealthy food products in retail food stores and BMI, dietary behaviors, purchasing, and sales of these foods?

#### Observational studies.

Of the 5 observational studies (n = 5/17; 29%) that assessed food positioning (Table 2), 3 (60%) ^51^^,^^55^^,^^56^^,^^60^^,^^63^showed that positioning strategies were consistently associated with outcomes beneficial to health (2 +s, 1 −ns).^55^^,^^56^^,^^60^ One of these studies assessed shelf positioning combined with store positioning and found results in the unexpected direction for shelf positioning (2 –ns) and inconsistent results for store positioning strategies (1 +ns, 1 −ns; separate results not shown).^63^

Of the 4 studies (n = 4/5; 80%) that assessed the positioning of healthy food products,^51^^,^^56^^,^^60^^,^^63^ 2 showed results in the expected direction (1 +s, 1 +ns)^56^^,^^60^ and 2 showed results in the unexpected direction (2 −ns).^51^^,^^63^ Additionally, 2 of these studies also assessed the positioning of unhealthy food items, with both showing results in the expected direction: Positioning unhealthy drinks and snacks at the ends of aisles, at checkouts, and in islands was associated with greater sales of these unhealthy items and increased BMI (1 +s, 1 +ns).^51^^,^^56^ Another study assessed only unhealthy food positioning and found significant results in the expected direction (+s).^55^

Sales and purchasing outcomes were reported in 4 of the studies (n = 4/5; 80%)^55^^,^^56^^,^^60^^,^^63^ that examined the positioning of food products; one study reported BMI,^51^ but no studies measured dietary outcomes. Three-quarters of studies (n = 3/4; 75%) reporting sales/purchasing outcomes showed that positioning healthy and unhealthy food products in prominent in-store locations was associated with greater sales of these products (2 +s, 1 +ns, 1 −ns).^55^^,^^56^^,^^60^ The single study that reported BMI revealed inconsistent results, with prominent positioning of both healthy and unhealthy foods showing associations with higher BMI.^51^

#### Intervention studies.

Twelve intervention studies^30^^,^^33–35^^,^^39^^,^^40^^,^^42^^,^^45–48^ (n = 12/22; 55%) described the effects of manipulating the positioning of food products, of which 4 (n = 4/12; 33%) included additional intervention components such as signage, social media campaign, and staff training.^33^^,^^40^^,^^47^^,^^48^ Of the 8 studies^30^^,^^34^^,^^35^^,^^39^^,^^42^^,^^45^^,^^46^ (n = 8/12; 75%) that tested only product positioning, 4 showed results in the expected direction for health (3 +s, 1 +ns)^30^^,^^35^^,^^39^ and 4 showed inconsistent or unexpected results.^34^^,^^42^^,^^45^^,^^46^ Two studies tested alternating treatment and control conditions. Treatment conditions included prominent positioning of healthy products alone or prominent positioning alongside point-of-purchase signage. The results were inconsistent for the positioning-alone strategies, but consistent positive results (2 +ns) were observed for the multicomponent condition.^47^^,^^48^ Of the studies describing the effects of prominent store positioning^30^^,^^33^^,^^34^^,^^39^^,^^40^^,^^42^^,^^45–48^ (n = 11/12; 92%), the majority (n = 6/11; 55%) showed these interventions have positive effects for health (3 +s, 3 +ns).^30^^,^^33^^,^^39^^,^^40^^,^^48^ The 2 studies (n = 2/12; 17%) that investigated the effects of shelf positioning, however, had inconsistent results (1 +ns, 1 −ns).^34^^,^^35^

Most studies (n = 5/9; 56%) that concurrently positioned healthy foods in prominent locations and unhealthy foods in less prominent locations showed results in the expected direction for health (2 +s, 3 +ns).^30^^,^^33^^,^^40^^,^^48^ Positioning healthy foods in more prominent locations led to healthier dietary-related outcomes in the majority (n = 3/5; 60%) of studies (2 +s, 1 +ns, 1 −ns, 1 inconsistent).^34^^,^^35^^,^^39^^,^^47^^,^^48^ The single study that altered the positioning of unhealthy food found that locating high-fat dairy products in a less prominent shelf position resulted in a nonsignificant decrease in sales of these items.^35^

Most studies (n = 7/12; 58%) reporting sales/purchasing outcomes showed that positioning products in prominent locations increased sales/purchases of these products (3 +s, 4 +ns).^30^^,^^33^^,^^35^^,^^39^^,^^40^^,^^48^ One study showed a decrease in eligible food sales relating to women, infants, and children after these products were positioned in prominent store and shelf locations.^34^ The single study (n = 1/12; 8%) measuring dietary outcomes showed that simultaneously placing fruit and vegetables at the front of the store, and crisps at the back, resulted in a nonsignificant increase in daily fruit and vegetable intake among intervention store customers compared with control customers.^40^

Availability and positioning were combined in 6 intervention studies.^27^^,^^31^^,^^32^^,^^38^^,^^41^^,^^49^ The vast majority of these studies (n = 5/6; 83%) showed results in the expected direction for health (5 +ns, 1 inconsistent).^31^^,^^32^^,^^38^^,^^41^^,^^49^ These results were consistent regardless of whether the intervention targeted healthy, unhealthy, or both types of products, or measured sales/purchases or dietary outcomes. Five studies reported findings from multicomponent interventions incorporating other strategies such as shelf labels, food demonstrations, and promotional events. The majority of these multicomponent intervention studies (n = 4/5; 80%) showed results in the expected direction for health (4 +ns, 1 inconsistent).^27^^,^^31^^,^^32^^,^^38^^,^^41^

### Research question 3: Do these associations differ according to socioeconomic position?

#### Observational studies.

Seven observational studies (41%) provided no description of the socioeconomic backgrounds of the study area or study participants. Of the 10 (59%) that reported socioeconomic data, 5 were conducted in study areas with varying levels of socioeconomic position (SEP)^55^^,^^57^^,^^58^^,^^63^^,^^65^ and 5 were conducted with participants of lower SEP or in areas of lower SEP.^51^^,^^53^^,^^59^^,^^62^^,^^64^ Only one study had an inclusion criterion that specifically targeted low-income participants.^53^ No observational studies explicitly examined the interaction between SEP and food placement strategies. However, from the studies conducted amongst predominantly disadvantaged groups (ie, low income, high prevalence of government assistance, or deprived area), findings showed consistently that healthier placement strategies were associated with better diet and sales outcomes, but the association with BMI outcomes was inconsistent.

#### Intervention studies.

Fifteen intervention studies (71%) described the socioeconomic backgrounds of the study area or study participants. Twelve of the studies (n = 12/15; 80%) reporting socioeconomic data were conducted in deprived neighborhoods, and 3 (n = 3/15; 20%) among populations of varying SEP. Only one study specifically analyzed the differential intervention effects according to household social class (occupation of highest earner) and found no clear trend across social class quintiles.^42^ In the 12 studies that focused on populations of lower SEP, half showed results indicating that the intervention was beneficial for health. The other half of the studies, however, showed inconsistent results.

### Risk of bias

Tables S5 and S6 (please see the Supporting Information online) present the risk-of-bias assessment results for each article.

#### Observational studies.

Nine observational studies (59%) were found to have a low risk of bias in relation to the research questions^52^^,^^53^^,^^59–63^^,^^65^^,^^66^; 6 (35%) were classified as having moderate risk of bias^51^^,^^55–58^^,^^64^ and 2 (12%) as having high risk of bias.^50^^,^^54^ Of the 9 classified as having a low risk of bias, 5 showed positive results (4 +ns,^52^^,^^59–61^ 1 +s^62^), 3 inconsistent results,^63^^,^^65^^,^^66^ and 1 negative results (−ns).^53^

#### Intervention studies.

Twenty (91%) intervention studies were classified as having a high risk of bias in relation to the research question,^27^^,^^30^^,^^31^^,^^34–36^^,^^38–49^and 2 (9%) had moderate risk of bias.^32^^,^^33^ The 2 studies with moderate risk of bias both showed results indicating that health product placement interventions can be beneficial for health; however, the results from these studies did not reach statistical significance.^32^^,^^33^

## DISCUSSION

### Summary of findings

This systematic review is the first to consider the overall direction of effect for food placement strategies on healthy eating behaviors. Considering the need for action on the complex public health concerns of poor diet and obesity, and the difficulties in conducting randomized controlled trials in this research field, this systematic review finds moderate evidence using a practice-based evidence perspective,^67^ from both observational and intervention studies, for food placement strategies in food retail stores positively influencing healthy eating behaviors. This review indicates weaker, but still meaningful, evidence of an effect when adopting the more traditional evidence-based practice approach. Although the majority of findings showed that greater availability and more prominent positioning of healthy foods, or reduced availability and less prominent positioning of unhealthy foods, related to better dietary behaviors, many were not statistically significant. The small sample size and lack of power demonstrated in many of the studies, particularly the intervention studies, may have contributed to the high number of nonsignificant results, and likely indicate the difficulties in conducting these types of field studies with a high-quality scientific design.

Analyzing the results with greater granularity, according to placement type the literature reveals moderate observational evidence for an association between product availability and dietary-related outcomes in the expected direction for health; evidence from intervention research was more limited and equivocal. A large proportion of both observational and intervention literature focused on improving the availability of healthy foods; hence, drawing conclusions on the effectiveness of solely limiting the availability of unhealthy foods is not yet possible. Both observational and intervention literature indicated moderate evidence for product positioning strategies in food stores affecting dietary-related health outcomes; most intervention studies indicated that more prominent positioning of healthy foods, and less prominent positioning of unhealthy foods, results in better dietary, or healthier sales, behaviors. Good evidence from the intervention studies included in this review exists to support strategies that combine the availability and positioning of both healthy and unhealthy foods to provide benefit for health. A number of interventions were multicomponent, which does somewhat weaken the conclusions we can draw about the effect of availability and positioning measures alone; however, the majority (80%) of the multicomponent interventions in this study showed findings in the expected direction for health. Sales outcomes, which were assessed in the majority of intervention studies and in almost half of the observational studies, provided the most consistent results in the expected direction for health benefit. The least abundant and least consistent evidence was found for BMI outcomes; this is perhaps not surprising given the multiple determinants of body weight and the challenges of assessing BMI in large-scale studies.

Observational studies suggested that strategies to improve the placement of healthy foods, and limit the placement of unhealthy foods, could have a positive impact on diet and sales in populations of low socioeconomic status. However, these results were not replicated among the intervention literature, which showed inconsistent findings in populations of low socioeconomic position.

### Policy implications

The results from this review provide policy makers with evidence to justify the implementation of population-level policies incorporating placement strategies in food retail stores to improve dietary-related behaviors. Although more research is needed to quantify the magnitude of effect of availability and positioning strategies, this review’s findings suggest that placement strategies combining both availability and positioning have the greatest potential to improve the healthfulness of sales and dietary patterns.

The evidence that is currently available – summarized in this review and the 2016 systematic review by Bucher et al,^68^ which found that manipulating the order and proximity of food in eateries and food service outlets influenced food choice – supports the UK government’s intention to ban the positioning of unhealthy food items in prominent locations in food retail stores and food service outlets.^69^ Other governments could also consider introducing similar policies to improve dietary quality across high-income countries. Even though the current research findings do not meet the “gold-standard” level of evidence that is usually required for the scientific community to provide certainty of effect, these reviews provide a sufficient body of evidence to recommend government action. The introduction of government policies to promote healthy food retail establishments would lead to a “level playing field” between retailers. If such placement strategies are only implemented on a voluntary basis, the high level of competition within this setting may result in some businesses not implementing such strategies, and that would likely limit the positive impact on public health.^70^

### Next steps for the research field

Although this review indicates moderate evidence for food placement strategies in retail food stores influencing purchasing and dietary behaviors, there are a number of ways in which the body of evidence can be strengthened. The lack of power calculations described in intervention research in particular is an issue that needs addressing to optimize external validity of the evidence. No intervention articles in this review described their power calculations or justified the study’s sample size. Placement studies require power calculations that take account of clustering at the store level because the intervention is store-based. In a cluster-designed study, it is the number of clusters, rather than the number of individuals within each cluster, that is most potent in determining statistical power.^71^ The need for a large number of stores and the opportunistic nature of many interventions studies are key reasons why no high-quality intervention research has been conducted in this field. Considerable commitment is required from commercial collaborators to allow for the required number of stores; however, mounting societal and political pressure for food retailers to engage in healthy eating strategies could enhance future prospects of adequately powered studies being conducted.

Improving the design of future food placement research, particularly considering novel trial designs and longitudinal observational studies, would further improve the evidence base. Less than two-thirds of the intervention articles in this review included a comparison group, and only 4 were randomized controlled trials. This finding indicates that store-based placement interventions do not easily conform to scientific gold standards. Researching in real-world settings, however, provides valuable knowledge to policy makers about intervention effectiveness in complex social contexts, particularly when studies are rigorously designed. Parallel designs with control groups matched on area characteristics and store sales (plus adjustment for confounders) offer a robust design and were used in approximately one-third of the current intervention evidence, all published in the last 5 years. Alternative designs including natural experiments, stepped-wedge designs, synthetic controls, and propensity scores could be further explored for use in future food placement intervention or policy evaluation studies.^72–75^

There is a gap in the evidence to describe how reducing the availability of unhealthy foods in food retail stores affects diet-related outcomes as most of the literature investigating placement strategies has focused on healthy foods. Although more challenging commercially than the “win-win” of targeting healthy foods in placement interventions,^45^ future research should focus on limiting the availability and prominent positioning of unhealthy foods – perhaps by replacing them with non-food items in an attempt to reduce overall food intake. Compensation to food retail chains for any loss in revenue may need to be considered. As highlighted in a recent scoping review, business outcomes of food retail strategies to improve health should be consistently reported in academic literature.^76^

There is also a need for greater harmonization of in-store assessment measures that act as exposures in observational research or fidelity assessments in intervention studies. Currently, 3 categories of availability measures are used: shelf space, variety, and composite scores. Considerable within-category variation exists, and the composite scores typically measure in-store factors such as price and quality in addition to availability. Positioning measures have focused almost entirely on prominent store locations such as checkouts, ends of aisles, and front of store; only one observational article measured shelf placement in this review. An in-store assessment tool that expands the existing validated GroPromo tool^55^ to include measures of availability and shelf placement would help to harmonize data in this field. Intervention research should include measures of both availability and positioning in their fidelity assessments because these two placement strategies are often intertwined. Positioning products at the checkouts or ends of aisles typically extends the availability of those products as they are located both in an aisle and in a prominent location. Only one intervention article in this review specifically considered both these aspects of placement in its evaluation.^47^

Consistent with the findings of previous reviews of supermarket interventions,^15^^,^^16^ many of the studies in this review contained multiple intervention components. Food placement strategies were a core component of these interventions. It was not possible, however, to decipher the isolated effects of changing product availability or positioning because additional strategies such as signage or staff training were employed at the same time. Future research that aims to test multiple intervention components, such as the 4 Ps of marketing, should consider the study by Wensel et al,^34^ which tested both the independent and additive effects of 4 intervention elements. This type of research, combined with studies that test single-component placement interventions, will be scientifically advantageous and ensure efficient and cost-effective packaging of interventions.

While sales outcomes were most frequently used and showed greatest consistency of effect in this review, loyalty card data were not used in any of the observational or intervention studies. Loyalty card data are a form of “big data” that offer a potentially economical method of analyzing how in-store determinants affect household purchasing.^16^^,^^77^ Little is known from the available literature about the populations who are most affected by placement strategies in food stores. This gap could be addressed by measuring intervention effects at a household or individual level rather than at a store level. Despite existing evidence suggesting that those from disadvantaged backgrounds are more susceptible, in dietary terms, to unhealthy in-store environments than those of more affluent groups,^78^ it is currently unclear whether placement strategies exacerbate or reduce dietary inequalities. Further evidence from adequately powered studies is needed to determine differential effects by SEP. Moreover, assessments of dietary outcomes, nutritional biomarkers or metabolites, and food waste are needed alongside loyalty card data to provide intelligence on the accuracy of this big data source, and the correlation between purchasing and intake patterns. It would be particularly useful if future research included dietary assessments from more than one household member to understand more clearly which household members are being affected by placement strategies. Finally, as outcome measures with frequent time points (weekly sales or purchasing data) become commonplace in this research field, more advanced statistical methods such as the time-series analyses used by Ejlerskov et al,^30^ and appropriate adjustment for clustering and confounding, should be more consistently applied.

### Strengths and limitations

This review is strengthened by the adherence to PRISMA guidelines throughout. Two reviewers independently conducted a risk-of-bias assessment and data extraction from each of the included studies to ensure consistency and rigor. In addition, the inclusion of both observational and intervention studies is a strength of this study as this allowed for a more thorough assessment of the overall relationship between placement strategies in food-store settings and diet-related outcomes. Product availability has been researched most extensively in the observational literature, while product positioning has been the focus of many intervention studies. However, including both types of literature presents some challenges with interpreting the quality of the evidence and summarizing overall results. Separate risk-of-bias assessment criteria were used for observational and intervention studies, so the final quality scores could not be compared. Intervention research is considered a higher grade of evidence and should be treated as such when drawing conclusions. This review also only included studies that assessed physical in-store environments, excluding virtual and online settings. This approach allows for the assessment of strategies that have greater external validity and the ability to be implemented in real-life settings. The findings of this review, however, are not applicable to the growing online grocery sales market.

The search strategy for this review did not include literature published prior to 2005 or forward searching for identified articles through citations. It is therefore possible that some articles of interest may have been excluded. However, 2005 marked the year a landmark article in the field of food environment research was published.^24^ This article, along with the Foresight obesity report,^3^ was among the first to highlight the importance of understanding the role of food environments on population health. Another limitation of this systematic review was the exclusion of gray literature or unpublished data in this topic area. Consequently, it is possible that the results of this review are subject to publication bias. Studies showing positive and significant effects may have an increased likelihood of publication, and our findings were potentially skewed as a result of this bias. In addition, owing to the heterogeneous nature of the study exposures, interventions, and outcomes, it is difficult to draw definitive conclusions from the available evidence. Though meta-analysis was not possible, a quantitative summary of the evidence was achieved by using a direction-based vote-counting technique or effect direction plot, as is recommended by Cochrane when meta-analysis is not feasible.^28^ This technique, however, is limited by its lack of consideration of the magnitude of effects and differences in study size.^28^

## CONCLUSIONS

Drawing on recent evidence from observational and intervention research across high-income countries, this review suggests that more prominent placement strategies are associated with higher sales and consumption of both healthy and unhealthy foods, but not weight status. Even though further high-quality research is required in this area, the balance of evidence suggests that the introduction of government interventions may be beneficial by providing a “level playing field” between retailers and to increase the availability and prominence of healthy foods and reduce the availability and prominent positioning of unhealthy foods. Future research priorities should focus on designing adequately powered intervention studies that test both the independent and additive effects of reducing the availability, and limiting the prominent positioning, of unhealthy foods. A greater understanding of who is most affected by placement strategies is required; this could be achieved through the use of loyalty card data as the primary outcome holds potential, alongside dietary assessments from more than one household member.

## Supplementary Material

nuaa024_Supplementary_Data
