# Supplementary material for: A systematic review of the influences of food store product placement on dietary-related outcomes
Source: Nutr Rev. 2020 Jun 1;78(12):1030–45. doi: 10.1093/nutrit/nuaa024 (PMC7666915; doi:10.1093/nutrit/nuaa024)
Supplement: nuaa024_Supplementary_Data [file nuaa024_supplementary_data.docx]

**Table S1: PRISMA Checklist**

| **Section/topic** | | **#** | | **Checklist item** | **Reported on page #** | |  |
| --- | --- | --- | --- | --- | --- | --- | --- |
| **TITLE** | | | | |  | |  |
| Title | | 1 | | Identify the report as a systematic review, meta-analysis, or both. | 1 | |  |
| **ABSTRACT** | | | | |  | |  |
| Structured summary | | 2 | | Provide a structured summary including, as applicable: background; objectives; data sources; study eligibility criteria, participants, and interventions; study appraisal and synthesis methods; results; limitations; conclusions and implications of key findings; systematic review registration number. | 2 | |  |
| **INTRODUCTION** | | | | |  | |  |
| Rationale | | 3 | | Describe the rationale for the review in the context of what is already known. | 4-5 | |  |
| Objectives | | 4 | | Provide an explicit statement of questions being addressed with reference to participants, interventions, comparisons, outcomes, and study design (PICOS). | 6 | |  |
| **METHODS** | | | | |  | |  |
| Protocol and registration | | 5 | | Indicate if a review protocol exists, if and where it can be accessed (e.g., Web address), and, if available, provide registration information including registration number. | 6 | |  |
| Eligibility criteria | | 6 | | Specify study characteristics (e.g., PICOS, length of follow-up) and report characteristics (e.g., years considered, language, publication status) used as criteria for eligibility, giving rationale. | Table 1 | |  |
| Information sources | | 7 | | Describe all information sources (e.g., databases with dates of coverage, contact with study authors to identify additional studies) in the search and date last searched. | 6 | |  |
| Search | | 8 | | Present full electronic search strategy for at least one database, including any limits used, such that it could be repeated. | Supp. Table 2 | |  |
| Study selection | | 9 | | State the process for selecting studies (i.e., screening, eligibility, included in systematic review, and, if applicable, included in the meta-analysis). | 6-7 | |  |
| Data collection process | | 10 | | Describe method of data extraction from reports (e.g., piloted forms, independently, in duplicate) and any processes for obtaining and confirming data from investigators. | 7 | |  |
| Data items | | 11 | | List and define all variables for which data were sought (e.g., PICOS, funding sources) and any assumptions and simplifications made. | 7 | |  |
| Risk of bias in individual studies | | 12 | | Describe methods used for assessing risk of bias of individual studies (including specification of whether this was done at the study or outcome level), and how this information is to be used in any data synthesis. | 7-8 | |  |
| Summary measures | | 13 | | State the principal summary measures (e.g., risk ratio, difference in means). | 8-9 | |  |
| Synthesis of results | | 14 | | Describe the methods of handling data and combining results of studies, if done, including measures of consistency (e.g., I^2^) for each meta-analysis. | 8-9 | |  |
| **Section/topic** | **#** | | **Checklist item** | | | **Reported on page #** | |
| Risk of bias across studies | 15 | | Specify any assessment of risk of bias that may affect the cumulative evidence (e.g., publication bias, selective reporting within studies). | | | 7-8 | |
| Additional analyses | 16 | | Describe methods of additional analyses (e.g., sensitivity or subgroup analyses, meta-regression), if done, indicating which were pre-specified. | | | N/A | |
| **RESULTS** | | | | | |  | |
| Study selection | 17 | | Give numbers of studies screened, assessed for eligibility, and included in the review, with reasons for exclusions at each stage, ideally with a flow diagram. | | | Figure 1 | |
| Study characteristics | 18 | | For each study, present characteristics for which data were extracted (e.g., study size, PICOS, follow-up period) and provide the citations. | | | Supp. Tables 5&6 | |
| Risk of bias within studies | 19 | | Present data on risk of bias of each study and, if available, any outcome level assessment (see item 12). | | | Sup. Tables 3&4 | |
| Results of individual studies | 20 | | For all outcomes considered (benefits or harms), present, for each study: (a) simple summary data for each intervention group (b) effect estimates and confidence intervals, ideally with a forest plot. | | | 17-21 | |
| Synthesis of results | 21 | | Present results of each meta-analysis done, including confidence intervals and measures of consistency. | | | 17-23 | |
| Risk of bias across studies | 22 | | Present results of any assessment of risk of bias across studies (see Item 15). | | | 23 | |
| Additional analysis | 23 | | Give results of additional analyses, if done (e.g., sensitivity or subgroup analyses, meta-regression [see Item 16]). | | | N/A | |
| **DISCUSSION** | | | | | |  | |
| Summary of evidence | 24 | | Summarize the main findings including the strength of evidence for each main outcome; consider their relevance to key groups (e.g., healthcare providers, users, and policy makers). | | | 23-29 | |
| Limitations | 25 | | Discuss limitations at study and outcome level (e.g., risk of bias), and at review-level (e.g., incomplete retrieval of identified research, reporting bias). | | | 29-30 | |
| Conclusions | 26 | | Provide a general interpretation of the results in the context of other evidence, and implications for future research. | | | 31 | |
| **FUNDING** | | | | | |  | |
| Funding | 27 | | Describe sources of funding for the systematic review and other support (e.g., supply of data); role of funders for the systematic review. | | | 32 | |

**Table S2: Search Strategy**

| **Database** | **Search Term** |
| --- | --- |
| Ovid MEDLINE(R) without Revisions | 1 fruit/ or nuts/ or seeds/  2 Vegetables/  3 (low adj fat).ti,ab.  4 (reduced adj fat).ti,ab.  5 (healthy or healthful).ti,ab.  6 (fresh adj produce).ti,ab.  7 (fresh adj food).ti,ab.  8 fruit.ti,ab.  9 vegetable*.ti,ab.  10 ((healthy or healthful) adj2 (diet* or food)).ti,ab.  11 nutritious.ti,ab.  12 1 or 2 or 3 or 4 or 5 or 6 or 7 or 8 or 9 or 10 or 11  13 exp Diet/  14 Feeding Behavior/  15 food preferences/  16 food habits/  17 13 or 14 or 15 or 16  18 12 or 17  19 (habit* or choice* or prefer*).ti,ab.  20 food.ti,ab,hw.  21 19 and 20  22 18 or 21  23 Food Supply/  24 Food Industry/  25 supermarket*.ti,ab.  26 (grocery adj1 store*).ti,ab.  27 (food adj1 store*).ti,ab.  28 (food adj1 retail*).ti,ab.  29 (retail adj1 store*).ti,ab.  30 food.ti,ab,hw.  31 29 and 30  32 outlet.ti,ab.  33 30 and 32  34 23 or 24 or 25 or 26 or 27 or 28 or 31 or 33  35 22 and 34  36 limit 35 to english language  **37 limit 36 to yr="2005 -Current"** |
| Scopus  Limits:  Health Sciences or Social Sciences and Humanities | ( ( ( ( TITLE ( ( ( healthy OR healthful ) W/2 ( diet* OR food* ) ) ) AND SUBJAREA ( mult OR medi OR nurs OR vete OR dent OR heal OR mult OR arts OR busi OR deci OR econ OR psyc OR soci ) AND PUBYEAR > 2004 ) OR ( ABS ( ( ( healthy OR healthful ) W/2 ( diet* OR food* ) ) ) AND SUBJAREA ( mult OR medi OR nurs OR vete OR dent OR heal OR mult OR arts OR busi OR deci OR econ OR psyc OR soci ) AND PUBYEAR > 2004 ) OR ( TITLE ( nutritious ) AND SUBJAREA ( mult OR medi OR nurs OR vete OR dent OR heal OR mult OR arts OR busi OR deci OR econ OR psyc OR soci ) AND PUBYEAR > 2004 ) OR ( ABS ( nutritious ) AND SUBJAREA ( mult OR medi OR nurs OR vete OR dent OR heal OR mult OR arts OR busi OR deci OR econ OR psyc OR soci ) AND PUBYEAR > 2004 ) OR ( ( TITLE ( fruit OR nuts OR seeds ) AND SUBJAREA ( mult OR medi OR nurs OR vete OR dent OR heal OR mult OR arts OR busi OR deci OR econ OR psyc OR soci ) AND PUBYEAR > 2004 ) OR ( ABS ( fruit OR nuts OR seeds ) AND SUBJAREA ( mult OR medi OR nurs OR vete OR dent OR heal OR mult OR arts OR busi OR deci OR econ OR psyc OR soci ) AND PUBYEAR > 2004 ) OR ( TITLE ( vegetables ) AND SUBJAREA ( mult OR medi OR nurs OR vete OR dent OR heal OR mult OR arts OR busi OR deci OR econ OR psyc OR soci ) AND PUBYEAR > 2004 ) OR ( ABS ( vegetables ) AND SUBJAREA ( mult OR medi OR nurs OR vete OR dent OR heal OR mult OR arts OR busi OR deci OR econ OR psyc OR soci ) AND PUBYEAR > 2004 ) OR ( TITLE ( low W/2 fat ) AND SUBJAREA ( mult OR medi OR nurs OR vete OR dent OR heal OR mult OR arts OR busi OR deci OR econ OR psyc OR soci ) AND PUBYEAR > 2004 ) OR ( ABS ( low W/2 fat ) AND SUBJAREA ( mult OR medi OR nurs OR vete OR dent OR heal OR mult OR arts OR busi OR deci OR econ OR psyc OR soci ) AND PUBYEAR > 2004 ) OR ( TITLE ( reduced W/2 fat ) AND SUBJAREA ( mult OR medi OR nurs OR vete OR dent OR heal OR mult OR arts OR busi OR deci OR econ OR psyc OR soci ) AND PUBYEAR > 2004 ) OR ( ABS ( reduced W/2 fat ) AND SUBJAREA ( mult OR medi OR nurs OR vete OR dent OR heal OR mult OR arts OR busi OR deci OR econ OR psyc OR soci ) AND PUBYEAR > 2004 ) ) OR ( ( ABS ( healthy OR healthful ) AND SUBJAREA ( mult OR medi OR nurs OR vete OR dent OR heal OR mult OR arts OR busi OR deci OR econ OR psyc OR soci ) AND PUBYEAR > 2004 ) OR ( TITLE ( "fresh produce" ) AND SUBJAREA ( mult OR medi OR nurs OR vete OR dent OR heal OR mult OR arts OR busi OR deci OR econ OR psyc OR soci ) AND PUBYEAR > 2004 ) OR ( ABS ( "fresh produce" ) AND SUBJAREA ( mult OR medi OR nurs OR vete OR dent OR heal OR mult OR arts OR busi OR deci OR econ OR psyc OR soci ) AND PUBYEAR > 2004 ) OR ( TITLE ( "fresh food" ) AND SUBJAREA ( mult OR medi OR nurs OR vete OR dent OR heal OR mult OR arts OR busi OR deci OR econ OR psyc OR soci ) AND PUBYEAR > 2004 ) OR ( ABS ( "fresh food" ) AND SUBJAREA ( mult OR medi OR nurs OR vete OR dent OR heal OR mult OR arts OR busi OR deci OR econ OR psyc OR soci ) AND PUBYEAR > 2004 ) OR ( TITLE ( fruit OR vegetable* ) AND SUBJAREA ( mult OR medi OR nurs OR vete OR dent OR heal OR mult OR arts OR busi OR deci OR econ OR psyc OR soci ) AND PUBYEAR > 2004 ) OR ( ABS ( fruit OR vegetable* ) AND SUBJAREA ( mult OR medi OR nurs OR vete OR dent OR heal OR mult OR arts OR busi OR deci OR econ OR psyc OR soci ) AND PUBYEAR > 2004 ) ) ) OR ( ( KEY ( diet ) AND SUBJAREA ( mult OR medi OR nurs OR vete OR dent OR heal OR mult OR arts OR busi OR deci OR econ OR psyc OR soci ) AND PUBYEAR > 2004 ) OR ( KEY ( feeding behavior ) AND SUBJAREA ( mult OR medi OR nurs OR vete OR dent OR heal OR mult OR arts OR busi OR deci OR econ OR psyc OR soci ) AND PUBYEAR > 2004 ) OR ( KEY ( food preferences ) AND SUBJAREA ( mult OR medi OR nurs OR vete OR dent OR heal OR mult OR arts OR busi OR deci OR econ OR psyc OR soci ) AND PUBYEAR > 2004 ) OR ( KEY ( food habits ) AND SUBJAREA ( mult OR medi OR nurs OR vete OR dent OR heal OR mult OR arts OR busi OR deci OR econ OR psyc OR soci ) AND PUBYEAR > 2004 ) ) ) OR ( ( TITLE-ABS-KEY ( food ) AND SUBJAREA ( mult OR medi OR nurs OR vete OR dent OR heal OR mult OR arts OR busi OR deci OR econ OR psyc OR soci ) AND PUBYEAR > 2004 ) AND ( ( TITLE ( ( habit* OR choice* OR prefer* ) ) AND SUBJAREA ( mult OR medi OR nurs OR vete OR dent OR heal OR mult OR arts OR busi OR deci OR econ OR psyc OR soci ) AND PUBYEAR > 2004 ) OR ( ABS ( ( habit* OR choice* OR prefer* ) ) AND SUBJAREA ( mult OR medi OR nurs OR vete OR dent OR heal OR mult OR arts OR busi OR deci OR econ OR psyc OR soci ) AND PUBYEAR > 2004 ) ) ) ) AND ( ( ABS ( food W/2 store* ) AND SUBJAREA ( mult OR medi OR nurs OR vete OR dent OR heal OR mult OR arts OR busi OR deci OR econ OR psyc OR soci ) AND PUBYEAR > 2004 ) OR ( ( KEY ( food supply ) AND SUBJAREA ( mult OR medi OR nurs OR vete OR dent OR heal OR mult OR arts OR busi OR deci OR econ OR psyc OR soci ) AND PUBYEAR > 2004 ) OR ( KEY ( food industry ) AND SUBJAREA ( mult OR medi OR nurs OR vete OR dent OR heal OR mult OR arts OR busi OR deci OR econ OR psyc OR soci ) AND PUBYEAR > 2004 ) OR ( TITLE ( supermarket ) AND SUBJAREA ( mult OR medi OR nurs OR vete OR dent OR heal OR mult OR arts OR busi OR deci OR econ OR psyc OR soci ) AND PUBYEAR > 2004 ) OR ( ABS ( supermarket ) AND SUBJAREA ( mult OR medi OR nurs OR vete OR dent OR heal OR mult OR arts OR busi OR deci OR econ OR psyc OR soci ) AND PUBYEAR > 2004 ) OR ( TITLE ( grocer* W/2 store* ) AND SUBJAREA ( mult OR medi OR nurs OR vete OR dent OR heal OR mult OR arts OR busi OR deci OR econ OR psyc OR soci ) AND PUBYEAR > 2004 ) OR ( ABS ( grocer* W/2 store* ) AND SUBJAREA ( mult OR medi OR nurs OR vete OR dent OR heal OR mult OR arts OR busi OR deci OR econ OR psyc OR soci ) AND PUBYEAR > 2004 ) OR ( TITLE ( food W/2 store* ) AND SUBJAREA ( mult OR medi OR nurs OR vete OR dent OR heal OR mult OR arts OR busi OR deci OR econ OR psyc OR soci ) AND PUBYEAR > 2004 ) ) ) AND ( LIMIT-TO ( LANGUAGE , "English" ) ) |
| PsycINFO | TI low N2 fat or AB low N2 fat  2 TI reduced N2 fat or AB reduced N2 fat  3 TI healthy or healthful or AB healthy or healthful  4 TI fresh N2 produce or AB fresh N2 produce  5 TI fresh N2 food or AB fresh N2 food  6 TI fruit or AB fruit  7 TI vegetable* or AB vegetable*  8 TI nutritious or AB nutritious  9 TI seeds or AB seeds  10 TI nuts or AB nuts  11 S1 OR S2 OR S3 OR S4 OR S5 OR S6 OR S7 OR S8 OR S9 OR S10  12 DE "Eating Behavior"  13 (DE "Eating Attitudes") OR (DE "Food Preferences")  14 DE "Diets"  15 S12 OR S13 OR S14  16 S11 OR S15  17 TI habit* or choice* or prefer* or AB habit* or choice* or prefer*  18 DE "Food" or TI food or AB food  19 S17 AND S18  20 S16 OR S19  21 TI supermarket* or AB supermarket*  22 TI grocer* or AB grocer*  23 TI greengrocer* or AB greengrocer*  24 TI hypermarket* or AB hypermarket*  25 TI food N1 store* or AB food N1 store*  26 TI retail* or AB retail*  27 TI food or AB food  28 S26 AND S27  29 TI food N1 outlet* or AB food N1 outlet*  30 S21 OR S22 OR S23 OR S24 OR S25 OR S28 OR S29  31 S20 AND S30  32 Limit to English |
| ScienceDirect | TITLE-ABSTR-KEY(fruit or nut or seed or vegetable or healthy or healthful or nutritious or fresh or diet or choice or choose or select or buy or purchase or prefer or habit) AND TITLE-ABSTR-KEY(point of purchase or checkout or tills or multi-buy or bundle or kiosk or layout or mix 'n' match or meal deal or shelf or aisle or bogof or get one free or display)  TITLE-ABSTR-KEY (Point of purchase or placement or tills or checkout or cashier or aisle or shelf or shelves or close or proximity or position or display or layout or presentation or marketing or strategy or impulse or promotion or promote or environment) AND TITLE-ABSTR-KEY (supermarket or food store or grocery store) |
| Cochrane Central Register of Controlled Trials | Supermarket  “Grocery store”  Food AND store  Food AND retail  Food AND outlet  food AND industry  (fruit* or nut* or seed* or vegetable* or healthy or healthful or nutritious or choice* or choos* or select* or buy or purchase* or fresh or prefer* or habit* or food or diet*) AND (supermarket or shop or outlet or retail or grocery or store) AND (placement or till or cashier or aisle or shelf or shelves or close or proximity or position or display or layout or presentation or marketing or strateg* or impulse or promotion or promote or environment) |
| Econlit (American Economic Association's electronic database) | (fruit* or nut* or seed* or vegetable* or healthy or healthful or nutritious or choice* or choos* or select* or buy or purchase* or fresh or prefer* or habit* or food or diet*) AND (supermarket or shop or outlet or retail or grocery or store) AND (placement or tills or cashier or aisle or shelf or shelves or close or proximity or position or display or layout or presentation or marketing or strateg* or impulse or promotion or promote or environment)  (behavior* or behaviour* or choice* or choos* or select* or buy or purchas* or habit* or prefer*) AND (placement or till or cashier or aisle or shelf or shelves or close or proximity or position or display or layout or presentation or marketing or strateg* or impulse or promotion or promote or environment) AND (supermarket or shop or outlet or retail or grocery or store) AND (fruit* or nut* or seed* or vegetable* or healthy or healthful or nutritious or fresh or food or diet*)  (supermarket or grocery or store) AND (fruit* or nut* or seed* or vegetable* or healthy or healthful or nutritious or fresh or food or diet*) AND (placement or till or aisle* or shelf* or shelv* or position or display or layout or presentation or marketing or strateg*) AND (behavior* or behaviour* or choice* or choos* or select* or buy or purchas* or habit* or prefer*)  (point of purchase or checkout* or tills or multi-buy* or bundle* or kiosk* or layout or mix 'n' match or meal deal* or shelf or aisle or bogof or get one free or display) AND (supermarket or food store or grocery store) |
| Cochrane Issue (Protocols for systematic reviews) | (food or diet*) AND (healthy or healthful or nutritious or fresh or fruit* or vegetable* or mediterranean)  Supermarket  Food AND store  Food AND retail  Food AND outlet  Food AND industry  food AND (choice* or habit* or prefer*) |
| Cochrane | Supermarket  “Grocery store”  Food AND store  Food AND retail  food AND outlet  food AND industry  food AND (choice* or habit* or prefer*)  (food or diet*) AND (healthy or healthful or nutritious or fresh or fruit* or vegetable* or mediterranean) |
| DARE | (fruit* or nut* or seed* or vegetable* or healthy or healthful or nutritious or choice* or choos* or select* or buy or purchase* or fresh or prefer* or habit* or food or diet*) AND (supermarket or shop or outlet or retail or grocery or store) AND (placement or till or cashier or aisle or shelf or shelves or close or proximity or position or display or layout or presentation or marketing or strateg* or impulse or promotion or promote or environment) |
| NIHR journals library | Supermarket or supermarkets  Grocery  Food  Shop or shops  Retail  outlet |

**Table S3: Quality Assessment Grading for Observational Studies**

| **Quality Assessment Criteria** | | | | | | | | | | | | | | | |
| --- | --- | --- | --- | --- | --- | --- | --- | --- | --- | --- | --- | --- | --- | --- | --- |
| **Observational Study** |  | **Study Design** | **Sample Description** | **Store Description** | **Exposure Measurement** | **Outcome** | **Blinding** | **Follow- up** | **Non- Participants** | **Variability** | **Analytical Methods** | **Confounding** | **Sample Size** | **Funding and Conflicts** | **Overall Risk of Bias*** |
|  | Bodor et al. (2008) ^S1^ | 0 | 0 | 0 | 0 | -1 | 0 | 0 | 0 | 1 | 1 | 1 | 0 | 1 | **Low** |
|  | Caldwell et al. (2009) ^S2^ | -1 | -1 | -1 | 0 | 0 | 0 | -1 | -1 | 1 | 1 | 0 | -1 | 1 | **High** |
|  | Caspi et al. (2017) ^S3^ | 1 | 1 | 0 | 0 | 0 | 0 | 0 | 0 | 0 | 1 | 1 | 0 | 1 | **Low** |
|  | Cohen et al. (2015) ^S4^ | 1 | 0 | 0 | 0 | 1 | 0 | 0 | 1 | -1 | -1 | -1 | 0 | 0 | **Moderate** |
|  | Franco et al. (2009) ^S5^ | 0 | 1 | -1 | 1 | 0 | 0 | 0 | 1 | 1 | 1 | 1 | 0 | 1 | **Low** |
|  | Gustafson et al. (2011) ^S6^ | 1 | 1 | -1 | 0 | 0 | 0 | 0 | 0 | 1 | 1 | 1 | 0 | 1 | **Low** |
|  | Gustafson et al. (2013) ^S7^ | 0 | 1 | 0 | 1 | 0 | 0 | 0 | 0 | 1 | 1 | 0 | 0 | 1 | **Low** |
|  | Jani et al. (2018) ^S8^ | -1 | -1 | -1 | -1 | -1 | 0 | 0 | -1 | -1 | -1 | 0 | -1 | 1 | **High** |
|  | Jilcott Pitts et al. (2017) ^S9^ | 1 | 1 | 0 | 1 | 1 | 0 | 0 | -1 | 1 | 1 | 1 | 0 | 1 | **Low** |
|  | Kerr et al. (2012) ^S10^ | 0 | -1 | 0 | 0 | 1 | -1 | 0 | 0 | 0 | 1 | -1 | 0 | -1 | **Moderate** |
|  | Martin et al. (2012) ^S11^ | 0 | 1 | 1 | 1 | -1 | 0 | 0 | 1 | 1 | 1 | 1 | 0 | 1 | **Low** |
|  | Nakamura et al. (2014)  ^S12^ | 0 | -1 | 0 | 0 | 1 | 0 | 0 | -1 | 1 | 1 | 1 | -1 | 0 | **Moderate** |
|  | Rose et al. (2009)  ^S13^ | -1 | 1 | 0 | 0 | 0 | 0 | 0 | 0 | 1 | 1 | 1 | 1 | 1 | **Low** |
|  | Ruff et al. (2016)  ^S14^ | 1 | 1 | 0 | -1 | 0 | 0 | 0 | 0 | 1 | 1 | 1 | 1 | 0 | **Low** |
|  | Sanchez-Flack et al. (2017)  ^S15^ | 1 | 1 | 1 | 1 | -1 | 0 | 0 | -1 | 1 | 1 | 1 | 0 | 1 | **Moderate** |
|  | Thornton et al. (2010)  ^S16^ | 0 | 1 | -1 | 0 | 0 | 0 | 0 | -1 | 1 | 0 | -1 | 1 | 1 | **Moderate** |
|  | Thornton et al. (2011)  ^S17^ | 0 | -1 | -1 | 0 | -1 | 0 | 0 | -1 | 1 | 1 | 1 | 1 | 0 | **Moderate** |
| *-1= Poor Quality; 0=Medium Quality; 1= High Quality*  ****Overall Risk of Bias: Observational Studies***- ≥5 Poor=High risk of bias, 2-4 Poor= Moderate risk of bias, ≤1 Poor= Low risk of bias | | | | | | | | | | | | | | | |

| **Quality Assessment Criteria** | | | | | | | | | | | | | | | | | | | |
| --- | --- | --- | --- | --- | --- | --- | --- | --- | --- | --- | --- | --- | --- | --- | --- | --- | --- | --- | --- |
| **Intervention Studies** |  | **Study Design** | **Randomisation** | **Assessor Blinding** | **Participant Blinding** | **Baseline Similarity** | **Selection Criteria** | **Participant Recruitment** | **Follow- up** | **Completers vs non completers** | **Drop out reasons** | **Outcome measurement** | **Intervention Integrity** | **Variability of Measures** | **Analytical Methods** | **Confounding** | **Sample Size** | **Funding and Conflicts** | **Overall Risk of Bias*** |
|  | Adam et al. (2017) ^S18^ | 0 | -1 | 0 | 0 | -1 | -1 | -1 | 1 | 0 | 1 | 1 | 0 | 1 | 1 | 1 | -1 | -1 | **High** |
|  | Adjoian et al. (2017)  ^S19^ | -1 | -1 | -1 | -1 | -1 | 0 | -1 | 0 | 0 | 1 | 0 | -1 | -1 | 0 | -1 | -1 | 0 | **High** |
|  | Albert et al. (2017)  ^S20^ | 0 | -1 | -1 | 0 | 0 | 0 | -1 | -1 | -1 | -1 | -1 | 0 | 0 | 0 | -1 | -1 | 1 | **High** |
|  | Ayala et al. (2013)  ^S21^ | 1 | -1 | -1 | 0 | -1 | 1 | 1 | -1 | 0 | 1 | 0 | 0 | 1 | -1 | -1 | -1 | 0 | **High** |
|  | Dannefer et al.  (2012)  ^S22^ | -1 | -1 | -1 | -1 | -1 | 0 | 0 | -1 | -1 | -1 | -1 | -1 | -1 | -1 | -1 | -1 | 0 | **High** |
|  | De Wijk et al.  (2016)  ^S23^ | -1 | -1 | -1 | 0 | -1 | -1 | -1 | 1 | 0 | 1 | 1 | 1 | -1 | 0 | -1 | -1 | 0 | **High** |
|  | Ejlerskov et al. (2018a1)  ^S24^ | 0 | -1 | -1 | 0 | -1 | -1 | 0 | -1 | 0 | -1 | 0 | -1 | 1 | 1 | 0 | 0 | 1 | **High** |
|  | Ejlerskov et al. (2018a2)  ^S24^ | 0 | -1 | -1 | 0 | -1 | -1 | -1 | -1 | -1 | -1 | 0 | -1 | 1 | 1 | 0 | 0 | 1 | **High** |
|  | Ejlerskov et al. (2018b)  ^S25^ | 0 | -1 | -1 | 0 | -1 | -1 | 0 | -1 | 0 | -1 | 0 | -1 | 1 | 1 | 1 | 0 | 1 | **High** |
|  | Foster et al.  (2014)  ^S26^ | 1 | 1 | -1 | 0 | -1 | 1 | 0 | 1 | 0 | 1 | 1 | 0 | 1 | 1 | 0 | -1 | 0 | **Moderate** |
|  | Gittelsohn et al.  (2010)  ^S27^ | 0 | -1 | -1 | 0 | -1 | 0 | -1 | -1 | 1 | 0 | -1 | 0 | 0 | 1 | 0 | 0 | 0 | **High** |
|  | Holmes et al.  (2012)  ^S28^ | -1 | -1 | -1 | -1 | -1 | -1 | -1 | 1 | 0 | 1 | 1 | 0 | -1 | 0 | -1 | -1 | 0 | **High** |
|  | Jilcott Pitts et al. (2018)  ^S29^ | 0 | -1 | -1 | 0 | -1 | -1 | -1 | -1 | -1 | 0 | 1 | -1 | 0 | 1 | 0 | -1 | 1 | **High** |
|  | Lawman et al.  (2015)  ^S30^ | -1 | -1 | -1 | -1 | -1 | 0 | 1 | -1 | -1 | -1 | 0 | -1 | 0 | 1 | -1 | 0 | 0 | **High** |
|  | Sigurdsson et al. (2009)  ^S31^ | -1 | -1 | -1 | -1 | -1 | -1 | -1 | 1 | 0 | 1 | 1 | 0 | 0 | -1 | -1 | -1 | -1 | **High** |
|  | Sigurdsson et al. (2011)  ^S32^ | -1 | -1 | -1 | -1 | -1 | -1 | -1 | 1 | 0 | 1 | 1 | 0 | 0 | -1 | -1 | -1 | 0 | **High** |
|  | Sigurdsson et al. (2014)  ^S33^ | -1 | -1 | -1 | -1 | -1 | -1 | -1 | 1 | 0 | 1 | 1 | -1 | -1 | -1 | -1 | -1 | 0 | **High** |
|  | Song et al. (2009)  ^S34^ | 0 | -1 | -1 | 0 | 0 | -1 | -1 | 1 | 0 | 1 | -1 | -1 | 1 | 0 | -1 | -1 | 1 | **High** |
|  | Thorndike et al.  (2017)  ^S35^ | 1 | -1 | -1 | 0 | -1 | 1 | 0 | 1 | 0 | 1 | 1 | 0 | 0 | 1 | 0 | -1 | 1 | **Moderate** |
|  | Toft et al. (2017)  ^S36^ | 0 | -1 | -1 | 0 | -1 | -1 | -1 | 1 | 0 | 1 | 1 | 0 | 1 | 1 | 0 | -1 | 1 | **High** |
|  | Wensel et al. (2019)  ^S37^ | 1 | -1 | -1 | 0 | -1 | 1 | 0 | 0 | 0 | 0 | -1 | 0 | 0 | 0 | -1 | -1 | 1 | **High** |
|  | Winkler et al.  (2016)  ^S38^ | -1 | -1 | -1 | -1 | -1 | -1 | -1 | 1 | 0 | 1 | -1 | -1 | 1 | 1 | 0 | -1 | 1 | **High** |
| *-1= Poor Quality; 0=Medium Quality; 1= High Quality*  ****Overall Risk of Bias: Intervention studies***: ≥6 Poor= High risk of bias, 3-5 Poor= Moderate risk of bias, ≤2 Poor= Low risk of bias | | | | | | | | | | | | | | | | | | | |

**Table S4: Quality Assessment Grading for Intervention Studies**

**Table S5: Summary Table of Observational Studies**

| **Author, Year, Country** | **Study Design** | **Setting** | **Participant**  **Sample** | **Placement Strategy** | **Exposure**  **Variable** | **Outcome** | **Key Findings** | **Result Summary *** | **Risk of Bias** |
| --- | --- | --- | --- | --- | --- | --- | --- | --- | --- |
| Bodor et al. (2008) ^S1^  USA | Cross-sectional | 15 convenience stores within 100m distance of participants’ homes in 4 contiguous census tracts  Deprived neighbourhoods in central New Orleans | n=102  Recruitment: Participants from random sample of households  Age: 82% aged 32+ years  Sex: 73% Female  Ethnicity: 37% White  53% Black  9% Other  Income: 31% below poverty threshold | Availability | **A:** Linear shelf space (m) of:   1. Fresh F 2. Total F (fresh, canned, frozen) 3. Fresh V 4. Total V (fresh, canned and frozen)   **A_V_:** Total number of F&V varieties  No details provided about when in-store audits were conducted | Total daily serves of F  Total daily serves of V  24-hour telephone administered recall using list of commonly consumed F&V, collected in 2001 | An increase in fresh F shelf space (m) within 100m of home, was associated (non-significant) with F intake (β 0.09; SE 0.11; p=0.43)  An increase in total F shelf space (m) within 100m of home, was associated (non-significant) with F intake (β 0.09; SE 0.07; p=0.23)  An increase in fresh F varieties within 100m of home, was associated (non-significant) with F intake (β 0.07; SE 0.09; p=0.41)  An increase in fresh V shelf space (m) within 100m of home, was associated (significant) with V intake (β 0.35; SE 0.16; p=0.03)  An increase in total V shelf space (m) within 100m of home, was associated (non-significant) with V intake (β 0.09; SE 0.05; p=0.07)  An increase in fresh V varieties within 100m of home, was associated (non-significant) with V intake (β 0.23; SE 0.12; p=0.06) | 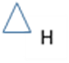  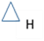  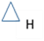  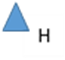  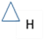  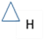 | Low |
| Caldwell et al.  (2009) ^S2^  USA | Longitudinal Survey  (Post hoc analysis as part of existing study)  BL  FU^1:^ 4-16 weeks  FU^2^: 12 months | 9 Colorado communities with one+ supermarkets  No neighbourhood SES details | n= 130  Recruitment: Participants of Colorado Healthy People 2010 Initiative living in 1 of the 9 assessed communities  No demographics for post-hoc analysis | Availability | **A:** Total metres^2^ of fresh F&V shelf space  **A:** Total linear metres of frozen, canned and juiced F&V shelf space.  **A_V_:** Total number of varieties of fresh F&V available in store e.g. 5 different types of apple – each counted as different variety.  No details provided about when in-store audits were conducted | Self-reported F&V consumption in past 7 days (portions).  6 questions about F&V and F juice taken from the Youth Risk Behaviour Survey | Greater availability (m^2^) of fresh F&V was associated with increased F&V consumption from BL to FU^1^ (β 0.02; CI95% 0.00, 0.04; p=0.01) and BL to FU^2^ (β 0.01; CI95% 0.00, 0.03; p=0.16)  Increased availability (m) of frozen, canned and juiced F&V was associated with increased F&V consumption from BL to FU^1^ (β 0. 46; CI95% 0.12, 0.80; p=0.01) and BL to FU^2^ (β 0. 12; CI95% -0.22, 0.46; p=0.47)  Increased number of varieties of fresh F&V was associated with increased F&V consumption from BL to FU^1^ (β 0.04; CI95% 0.01, 0.07; p=0.01) and BL to FU^2^ (β 0.02; CI95% -0.01, 0.05; p=0.15) | 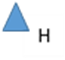  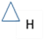  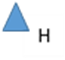  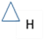  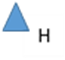  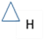 | High |
| Caspi et al.  (2017) ^S3^  USA | Cross-sectional | 99 convenience stores  Minnesota  No neighbourhood SES details reported | n=594  Recruitment: Convenience sample of shoppers exiting stores and participated in interview  Age (mean): 40 years  Sex: 58% male  Ethnicity: 3% Hispanic  48% White  36% Black  Employed: 64% | Availability  Position | **A:** Availability (Healthy Food Supply Score (HFS) using Yale Rudd Centre Tool) for 69 foods.  [HFS score (possible range 0-31) summarises availability, quality, variety and price of food in store. Higher scores represent healthier stores.]  **A:** Weight (lb) of F&V and wholegrain products for which standard items weights multiplied by counts of items  **A:** Linear shelf space (ft) for:   1. F&V 2. SSBs 3. Salty snacks   **A:** Shelf space Ratio F&V: SSBs and salty snacks  **Av:** Number of varieties of fresh, frozen and canned F&V and wholegrain products  **P:** Healthy food items found at checkout (Yes/ No)  **P:** F&V seen from front of the store (Yes/ No)  In-store audits conducted between Jul-Nov 2014 | Healthy Eating Index-2010 Score (0-100) for customer purchases. Higher scores indicate greater compliance with healthy eating guidelines.  Proportion of customers purchasing at least 1 F&V portion  Proportion of customers purchasing at least 1 wholegrain item.  Customer purchase assessment conducted when exiting the store. | Higher store HFS scores were associated with higher customer HEI-2010 (β 0.2; SE 0.2; p=0.2)  Customers of stores with high shelf space of F&V had higher mean customer HEI-2010 scores when compared to store with no and low F&V shelf space (Mean HEI score: High 35.2; Low 30.8, None 28.8; p for trend<0.01)  Customers of stores with a higher shelf space ratio of F&V: unhealthy beverages and snacks had higher mean customer HEI-2010 scores when compared to store with low ratios (Mean HEI score: High 35.9; Low 30.2, None 28.8; p for trend<0.01)  Customers had greater odds (non-significant) of purchasing at least 1 portion of F&V if stores stocked ≥90 lbs of fresh and frozen F&V compared to those selling 1-29 lbs (OR 3.0; CI95% 0.9, 9.9; p>0.05)  Customers had greater odds (non-significant) of purchasing at least 1 wholegrain item if stores stocked ≥30 lbs of wholegrains compared to those selling 0.4 lbs (OR 1.9; CI95% 0.5, 6.0; p>0.05)  Customers had greater odds (non-significant) of purchasing at least 1 portion of F&V if stores had higher shelf space of fresh F&V (OR 2.1; CI95% 0.7, 5.8; p>0.05)  Customers had greater odds (non-significant) of purchasing at least 1 portion of F&V if stores had a higher shelf space ratio of F&V: Unhealthy drinks and salty snacks (OR 2.7; CI95% 0.9, 7.9; p>0.05)  Customers had greater odds (significant) of purchasing at least 1 portion of F&V if stores stocked ≥14 varieties of F&V compared to those selling <7 varieties (OR 3.9; CI95% 1.2, 12.3; p<0.05)  Customers had greater odds (non-significant) of purchasing at least 1 wholegrain item if stores stocked ≥4 varieties of wholegrains compared to those selling 0-1 varieties (OR 1.1; CI95% 0.3, 3.8; p<0.05)  Customers had greater odds (non-significant) of purchasing at least 1 portion of F&V if stores had healthy food items at the checkout (OR 1.3; CI95% 0.6, 2.9; p<0.05)  Customers had greater odds (significant) of purchasing at least 1 portion of F&V if stores had fresh F&V visible from the entrance (OR 2.3; CI95% 1.0, 5.8; p<0.05) | 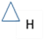  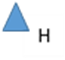  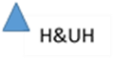  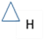  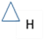  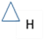  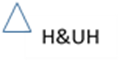  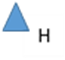  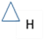  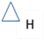  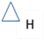 | Low |
| Cohen et al.  (2015) ^S4^  USA | Cross-sectional | 13 supermarkets  where participants reported shopping  2 low-income, majority African American neighbourhoods, Pittsburgh | n=980  Recruitment: Main food shopper in houses from stratified random sample of 2900 residential addresses from the study neighbourhoods  Age: >75% 40+ years  Sex: 73% Female  Ethnicity: 94% African American  Government benefits (SNAP): 50% | Position | **P:** Number of prominent store position displays (end-of-aisle, special floor and checkout) for:   1. SSBs 2. Unhealthy snack foods (confectionery, biscuits, cakes and salty snacks) 3. F&V and products with >50% whole grains   Final exposure variable= number of prominent position displays multiplied by frequency of visits to store, summed for all stores visited  In-store audits conducted May – Dec 2011 | BMI (kg/m^2^) - 84% objectively measured by research staff, others were self-reported.  2 interview administers 24-hour diet recalls completed and used to derive:   1. Healthy Eating Index score 2. Daily F&V portions 3. Daily sugar sweetened beverage intake (oz) | Greater exposure to sugar-sweetened beverage displays in prominent positions was associated with higher BMI (β 0.01/display, p=0.05)  Greater exposure to unhealthy snack foods in prominent positions was associated (non-significant) with higher BMI (β 0.000/display, p>0.05)  Greater exposure to F&V and wholegrain products in prominent positions was associated (non-significant) with higher BMI (β 0.00/display, p>0.05)  No CI95% reported | 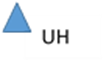  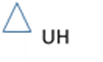  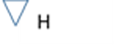 | Mod |
| Franco et al.  (2009) ^S5^  USA | Cross-sectional | n=226 supermarkets and convenience stores located in 159 contiguous census tracts located 1 mile from participants’ homes  Varying levels of neighbourhood deprivation, Baltimore City and Baltimore County | n=759  Recruitment: Participants in Baltimore arm of Multi-Ethnic Study of Atherosclerosis  Age (mean): 63 years  Sex: 52% Female  Ethnicity: 50% Black  50% White  Income: 18% Low | Availability | **A:** Continuous availability score (Standard deviation (SD)) (availability sub-component adapted NEMS-S) of healthier food items across 8 food categories for:   1. All stores in census tract 2. All stores in 1-mile radius 3. Closest store to home residence.   In-store audits conducted in 2006 | 2 dietary patterns:   1. Fat and processed meat pattern (poor quality) 2. Whole grains and F pattern (better quality)   Identified using principal component analysis on self-reported dietary data collected via 120-item FFQ for foods eaten in previous year collected between 2000-2002. | For each SD increase in healthy food availability in census tract, the poor quality dietary pattern score decreased (β -0.02; SE 0.04; p>0.05)  For each SD increase in healthy food availability in closest food store, the poor quality dietary pattern score decreased (β -0.05; SE 0.04; p>0.05)  For each SD increase in healthy food availability in all stores within 1 mile, the poor quality dietary pattern score decreased (β -0.01; SE 0.04; p>0.05)  For each SD increase in healthy food availability in census tract, the better quality dietary pattern score increased (β 0.03; SE 0.04; p>0.05)  For each SD increase in healthy food availability in closest food store, the better quality dietary pattern score decreased (β -0.00; SE 0.04; p>0.05)  For each SD increase in healthy food availability in all stores within 1 mile, the better quality dietary pattern score decreased (β -0.09; SE 0.04; p>0.05) | 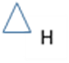  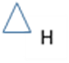  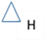  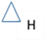  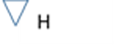  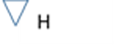 | Low |
| Gustafson et al. (2011) ^S6^  USA | Cross-sectional | 22 supermarkets identified by participants as being used for primary food shop  Small urban city, with majority white population, Lexington, Kentucky  No neighbourhood SES details reported. | n=121  Recruitment: Participants (n=1400) of a previous state-wide survey on cancer control and prevention sent a postal invitation    Age (mean): 42 years  Sex: 58% Females  Ethnicity: 94% White  2% African American  Income: 60% High (>= $50,000) | Availability | **A:** Availability score (adapted NEMS-S) for 55 healthier food items across 15 food categories  Data collected NEMS-S to derive a composite score incorporating availability, price and quality.  No details provided about when in-store audits were conducted | Binary variables for 4 food groups based on self-reported consumption:   1. F&V (<2 or ≥ 2 times per day) 2. High fibre cereals (<1 or ≥1 per day) 3. Sweetened beverages (never or ≥ 1 per week) 4. Biscuits and cakes (<5 or ≥5 per week)   26 items NHANES 2009-2010 dietary screener administered via telephone | Greater healthier food availability was associated with lower odds (non-significant) of consuming F&V (OR: 0.95; CI95% 0.83, 1.08; p>0.05)  Greater healthier food availability was associated with significantly lower odds of consuming sugar sweetened beverages (OR: 0.65; CI95% 0.14, 0.83; p≤0.05)  Greater healthier food availability was associated with lower odds (non-significant) of consuming biscuits and cakes (OR: 0.94; CI95% 0.38, 2.39; p>0.05)  Greater healthier food availability was associated with greater odds (non-significant) of consuming high-fibre cereal (OR: 1.05; CI95% 0.93, 1.20; p>0.05) | 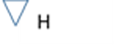  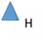  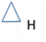  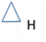 | Low |
| Gustafson et al. (2013) ^S7^  USA | Cross-sectional | 80 supermarkets identified by participants as being used for primary food shop  Primarily low-income and minority communities from urban and non-urban districts in North Carolina | n=186  Recruitment: Low-income women enrolled in weight loss intervention study  Age (mean): 51 years  Income: 69% low (<= $29,000) | Availability | **A:**  Availability score (adapted NEMS-S) ranging from 0-37 for 37 food items from 9 healthy food groups:   1. Non-fat/ low fat milk 2. F 3. V 4. Low-fat meats 5. Frozen F 6. Frozen V 7. Canned V 8. 100% whole wheat bread 9. Non-sugar sweetened cereal   Final score for each store categorised by tertiles into low, medium, high. Analyses compared high with low availability.  In-store audits conducted in spring and summer 2009 using availability subcomponent of NEMS-S | BMI (kg/m^2^ )- Objectively measured height and weight  Self-reported consumption F&V (portions per day)  Consumption data collected using the validated rapid food survey | High availability of healthy food, when compared with low availability, was associated (non-significant) with greater BMI (OR 1.13; CI95% -2.34, 4.47; p>0.05)  High availability of healthy food, when compared with low availability, was associated (non-significant) with lower consumption of F&V (OR 0.73; CI95% -0.77, 2.23; p>0.05) | 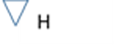  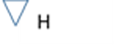 | Low |
| Jani et al.  (2018) ^S8^  New Zealand | Cross-sectional | 392 supermarkets  2 urban and 96 rural resident defined neighbourhood in Waikato/ Lake Districts.  No neighbourhood SES details reported. | n= 98 geographical neighbourhood boundaries for which obesity prevalence was calculated  Recruitment: Retrospective data from n= 3817 participants of the Te Wai o Rona: Diabetes prevention strategy study.  Ethnicity: 100% Polynesian (Māori) | Availability | **A:** The number of supermarkets within each neighbourhood where the healthier items was available as well as or instead of the regular item for all of the following items:   1. White vs wholemeal bread 2. Skin vs lean chicken 3. Regular vs trim meat 4. Whole milk vs skimmed milk 5. SSB vs water   **A:** Availability (adapted NEMS-S) of all 5 healthier alternative items listed above (Availability score ranging from 0-10)  In-store audits conducted in 2005 | Median obesity prevalence for neighbourhood defined as BMI ≥30kg/m^2^  BMI(kg/m^2^) - Objectively measured height and weight  Analyses adjusted for:   1. location (rural/urban) 2. median neighbourhood income | Greater number of supermarkets containing the healthy options available for all items was correlated with a non-significant decrease in BMI prevalence (r= -0.01, p= 0.99)  Higher healthy food availability score for all items was correlated with a non-significant increase in BMI prevalence (r= 0.01, p= 0.96) | 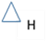  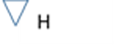 | High |
| Jilcott Pitts et al. (2017)^S9^  USA | Cross-sectional  Part of North Carolina Healthy Food Small Retailer Program | 16 convenience stores  North Carolina  No neighbourhood SES details reported. | n=479  Recruitment: Convenience samples of shoppers exiting store  Age (mean): 43 years  Sex: 41% Female  Ethnicity: 65% African American | Availability | **A**: Availability (Healthy Food Supply Score (HFS)) for 18 foods using adapted NEMS-S  [HFS score (possible range 0-31) summarises availability, quality, variety and price of food in store. Higher scores represent healthier stores]  No details provided about when in-store audits were conducted | Objective, non-invasive reflection spectroscopy (RS) to measure skin carotenoids, biomarker of F&V intake. Readings range from 0-800, with higher score indicating higher skin carotenoids and greater consumption of F&V  Self-report consumption of:   1. F&V portions / day 2. Soft drink/ day 3. Sweetened F drink/ day   Data collected using the National Cancer Institute F&V Screener | A positive association (non-significant) was observed between store level HFS score and skin carotenoids measured by RS device (β 2.21; CI95% -2.80, 7.21, p=0.38)  A negative (non-significant) association was observed between store HFS score and self-reported daily F&V consumption (β -0.01; CI95% -0.16, 0.13; p=0.86)  A negative (non-significant) association was observed for store HFS score and daily soft drink consumption (β -0.01; CI95% -0.08, 0.06; p=0.81)  A negative (non-significant) association was observed for store HFS score and daily sweetened F drink consumption (β -0.04; CI95% -0.09, 0.01; p=0.16) | 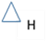  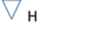  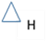  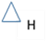 | Low |
| Kerr et al.  (2012)^S10^  USA | Cross-sectional | 37 supermarkets in 3 neighbourhoods  Varying levels of neighbourhood deprivation, San Diego | n= 637  Recruitment: Convenience sample of adult shoppers exiting study stores  No demographic characteristics provided | Position | **P:** Presence of food items from 7 food groups (crisps, confectionary, soft drinks, biscuits, F drinks and F&V) in store positions of high prominence:   1. Aisle ends near checkouts 2. Checkouts sides 3. Checkout ends   In-store audits conducted in 2007 using the GroPromo tool | % food dollars spent on:   1. Unhealthy Items (crisps, confectionary, soft drinks, biscuits and F drinks) 2. F&V   Data collected from customer receipts straight after in-store audits were conducted | Greater numbers of unhealthy items in high prominence areas was significantly associated with lower % of food dollars spent on F&V (β -0.7; p<0.01)  Greater numbers of unhealthy items in high prominence locations was significantly associated with higher % of food dollars spent on unhealthy products (β 0.41; p=0.04) | 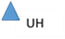  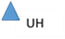 | Mod |
| Martin et al.  (2012)^S11^  USA | Cross-sectional | 19 convenience stores  Deprived areas with predominantly Hispanic and African American communities in Connecticut | n=372  Recruitment: Convenience sample of store customers approached before entering store  Age (mean): 38 years  Sex: 84% female  Ethnicity: 54% African American  40% Hispanic  6% Other  Government benefits (SNAP): 70% | Availability | **A_V_:** Number of varieties of:   1. Fresh F 2. Fresh V   In-store audits conducted in Jan-Feb 2009 using adapted NEMS-S | Probability of purchasing:   1. Fresh F 2. Fresh V   Self-reported purchasing data for the previous 3 months, collected by interviews in March-May 2009 | Increased in-store F variety was associated (significantly) with increased odds of customers purchasing F at the store (OR 1.12; CI95% 1.01, 1.25; p=0.03)  Increased in-store V availability was associated (significantly) with increased odds of customers purchasing F at the store (OR 1.15; CI95% 1.07, 1.23; p=0.01) | 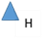  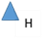 | Low |
| Nakamura et al.  (2014)^S12^  UK | Cross-sectional | One supermarket located in England  No neighbourhood SES details | N/A | Position | **P:** Dichotomise variable (present/ not present) for end of aisle displays of 3 drink products:   1. Carbonated Beverages 2. Tea 3. Coffee   In-store audit conducted for 13 weeks over a 1-year period from 2010-2011 by TNS PathTracker researchers | Mean weekly sales for 3 drink products  Objective store-level sales data collected by TNS PathTracker for same 13 weeks over a 1-year period from 2010-2011 | Carbonated beverages significantly greater in sales volume when placed at the end of aisle (β 0.42 CI95% 0.23, 0.61; p<0.01). Estimated increase (log transformed coefficient) = 51.7%  Coffee significantly greater in sales volume when placed at the end of aisle (β 0.55; CI95% 0.29, 0.82; p<0.01). Estimated increase (log transformed coefficient) = 73.5%  Tea significantly greater in sales volume when placed at the end of aisle (β 0.76; CI95% 0.22, 1.30; p<0.001). Estimated increase (log transformed coefficient) = 113.8% | 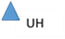  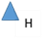  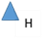 | Mod |
| Rose et al.  (2009) ^S13^  USA | Cross-sectional | 38 supermarkets,  119 convenience/ petrol stores, general merchandise and liquor stores in 103 census tracts  Urban areas, South-Eastern Louisiana.  No neighbourhood SES details | n=1243  Recruitment: Random sample of households contacted via telephone  Age: 63% aged <50 years  Sex: 66% Female  Ethnicity: 50% White  42% African American  5% Latino  3% Other | Availability | **A:** Total shelf space (m) in all food stores within 1km distance from participants’ homes for:   1. Total F&V 2. Total energy dense snack foods (confectionery, biscuits, salty snacks soft drink)   In-store audits conducted in 2004-2005 | BMI (kg/m^2^) - Self-reported weight and height  Data collected in 2004-2005 through telephone interview | Neighbourhood (within 500m of home) availability of F&V was not significantly associated with BMI (β 0.00; SE 0.00; p>0.05)  Neighbourhood (within 500m of home) availability of energy dense snack was significantly associated with BMI (β 0.00; SE 0.00; p>0.05)  Neighbourhood (within 1km of home) availability of F&V was not significantly associated with BMI (β 0.00; SE 0.00; p>0.05)  Neighbourhood (within 1km of home) availability of energy dense snack was significantly associated with BMI (β 0.00; SE 0.00; p<0.05)  Neighbourhood (within 2km of home) availability of F&V was not significantly associated with BMI (β -0.00; SE 0.00; p>0.05)  Neighbourhood (within 2km of home) availability of energy dense snack was significantly associated with BMI (β 0.00; SE 0.00; p>0.05) | 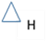  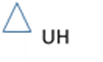  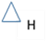  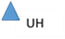  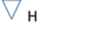  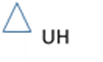 | Low |
| Ruff et al.  (2016) ^S14^  USA | Cross-sectional | 171 convenience stores (Bodegas)  Varying levels of neighbourhood deprivation, New York City | n= 1904  Recruitment: Convenience sample of shoppers exiting stores  Age: 61% 18-44 years  39% 45+ years  Sex: 45% Female  Ethnicity: 35% Black  29% Hispanic  18% White | Availability  Position | **Av:** Total number of individual types of fresh F&V available  **P:** Water placed at eye level (Yes/ No)  **P:** Fresh F&V displayed at front of store (Yes/ No)  No details provided about when in-store audits were conducted | Purchases of fresh F&V  Purchases of SSBs  Data recorded from bag checks when customers were exiting store | When compared to stores with high varieties of fresh F&V (11+ varieties), customers had decreased odds (significant) of purchasing fresh F&V if stores stocked fewer varieties of fresh F&V:   - 1 -5 types (OR 0.11; CI95% 0.04, 0.31; p<0.05) - 6 -10 types (OR 0.16; CI95% 0.06, 0.42; <0.05)   Customers had decreased odds (non-significant) of purchasing fresh F&V if the store did not have fresh F&V displayed at the front of the store (OR 0.827; CI95% 0.309, 2.214; p>0.05)  Customers had decreased odds (non-significant) of purchasing SSBs if the store did not have fresh F&V displayed at the front of the store (OR 0.745; CI95% 0.504, 1.102; p>0.05)  Customers had decreased odds (non-significant) of purchasing SSBs if the store did not have water displayed at eye level (OR 0.945; CI95% 0.72, 1.242; p>0.05)  Customers had decreased odds (non-significant) of purchasing fresh F&V if the store did not have water displayed at eye level (OR 0.784; CI95% 0.36, 1.73; p>0.05)  When compared to stores with 11+ varieties of fresh F&V, customers had greater odds of purchasing SSBs if stores stocked fewer varieties of fresh F&V:   - No varieties (OR 2.271; CI95% 1.274, 4.049; P<0.05) - 1-5 varieties (OR 1.788; CI95% 1.079, 2.963; P<0.05) - 6-10 varieties (OR 1.33; CI95% 0.777, 2.277; p>0.05) | 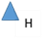  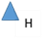  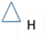    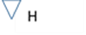  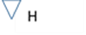  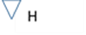  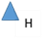  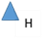  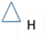 | Low |
| Sanchez-Flack et al. (2017)^S15^  USA | Cross-sectional | 16 convenience stores (Tiendas)  Located in areas with >20% Hispanic residents in San Diego County | n=356  Recruitment: Convenience sample of customers exiting stores  Age (mean): 42 years  Sex: 70% female  Ethnicity: 100% Hispanic  Employed: 60%  Government benefits (SNAP/ WIC): 48% | Availability | **A:** Total availability of 73 fresh, 28 canned and 16 frozen F&V, plus any others available. Scores summed to produce a continuous fresh F&V availability score and a continuous canned and frozen F&V availability score for each store  **A:** Total number of fresh F&V displays  **A:** Total shelf-space (feet^2)^ dedicated to fresh F&V  In-store audits conducted between November 2013 and October 2013 | Self-reported dollars spent on F&V per week at study store.  Data collected through store exit interviews with participants. | A positive association (significant) between the number of fresh F&V available and dollars spent on F&V per week in store (β 0.36; CI95% 0.09, 0.63; p=0.01)  A positive association (non-significant) between the number of canned and frozen F&V available and dollars spent on F&V per week in store (β 0.07; CI95% -0.14, 0.28; p=0.48)  When F&V shelf-space and number of F&V displays were considered in the same model, a positive association (significant) was observed between the number of F&V displays and dollars spent on F&V per week in store (β 0.02; CI95% 0.01, 0.04; p=0.01)  When F&V shelf-space and number of F&V displays were considered in the same model, a negative association (significant) was observed between F&V shelf-space (ft^2^) and dollars spent of F&V per week in store (β -0.29; CI95% -0.52, -0.06; p=0.02) | 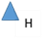  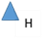  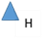  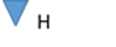 | Low |
| Thornton et al.  (2010) ^S16^  Australia | Cross-sectional | 143 supermarkets in 37 neighbourhoods  Random sample of neighbourhoods with varying levels of deprivation, Melbourne | n= 1082  Recruitment: Participants in the Social Status and Activity in Women study, living in a study neighbourhood which contained a supermarket within 3km of their home  Demographics of wider study (n=1399)  Age (mean): 41 years  Sex: 100% female  Neighbourhood SES: Low 33.2%  Mid 38.5%  High 28.3% | Availability | **A:** Availability of 15 commonly consumed F; summed to produce total F availability for each store  **A:** Availability of 23 commonly consumed V; summed to produce total V availability for each store  In-store audits conducted in 2006 using a structured checklist | Self-reported daily consumption of F&V portions; dichotomous outcome created for F&V separately:   1. High intakes= ≥2 portions of F/V daily 2. Low intakes= <2 portions of F/V daily   Data collected in 2004 via postal questionnaire adapted from the Australian National Nutrition Survey | A lower (non-significant) mean F availability was observed for customers who consumed ≥2 portions of F daily (10.5 (SD 2.2)) when compared to those who consumed <2 portions of F daily (10.6 (SD 2.1) (p=0.35)  A lower (non-significant) mean F availability was observed for customers who consumed ≥2 portions of V daily (10.5 (SD 2.2)) when compared to those who consumed <2 portions of V daily (10.7 (SD 2.1)) (p=0.190  A lower (non-significant) mean V availability was observed for customers who consumed ≥2 portions of F daily (20.3 (SD 4.0)) when compared to those who consumed <2 portions of F daily (20.5 (SD 3.8) (p=0.52)  A lower (significant) mean V availability was observed for customers who consumed ≥2 portions of V daily (20.2 (SD 4.1) when compared to those who consumed <2 portions of V daily (20.8 (SD 3.6)) (p=0.03) | 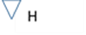  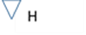  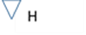  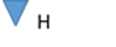 | Mod |
| Thornton et al.  (2011) ^S17^  Australia | Cross-sectional | 71 supermarkets in 35 neighbourhoods  Random sample of neighbourhoods with varying levels of deprivation, Melbourne | n=1007  Recruitment: Participants in the Social Status and Activity in Women study, living in one of the assessed neighbourhoods which contained a supermarket within 3km of their home  Demographics of wider study (n=1399)  Age (mean): 41 years  Sex: 100% female  Neighbourhood SES: Low 33.2%  Mid 38.5%  High 28.3% | Availability | **A_V_:** Mean number of varieties of chocolates for all supermarkets within 3km from home; categorical variable developed:  <40 items,  40-49 varieties,  ≥50 varieties    **Av:** Mean number of varieties of confectionery for all supermarkets within 3km from home; categorical variable developed:  <40 items,  40-49 varieties,  ≥50 varieties  In-store audits conducted in 2006 using a structured checklist | Self-reported consumption for previous month (1-3/ month, 1/ week, 2-6/ week, 1+/day)  of:   1. Chocolate 2. Confectionery   Data collected in 2004 via postal questionnaire adapted from the Australian National Nutrition Survey | For chocolate, greater variety was non-significantly associated with frequency of consumption of chocolate with OR’s very close to 1. Effect estimates were higher for daily consumption (ORs>1.4, p>0.3). However, ≥50 varieties were associated with lower odds of consumption 2-6 times per week (OR (95% CI): 0.71 (0.39-1.29), p=0.26).  For confectionary, greater variety was non-significantly associated with frequency of consumption with ORs very close to 1. Larger positive effect estimates were found for the association between variety and consumption 2-6 times per week (ORs>1.27, p>0.40). A variety of 40-49 was associated with higher monthly consumption (OR=1.31, p=0.35) and ≥50 varieties was associated with higher daily consumption (OR=1.46, p=0.48). | 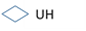  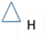 | Mod |

**Result summary:** Direction of result, significance level (**
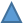
** Positive result p<0.05; **
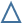
** Positive result p>0.05; **
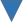
** Negative result p<0.05**;**
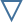
 Negative result p>0.05; Direction inconsistent) & health rating of exposure; **SES:** Socio-economic Status**; F:** Fruit**; V:** Vegetables**; F&V:** Fruit and vegetables; **WIC**: Women, Infants, Children; **SNAP**: Supplemental Nutrition Assistance Program; **SSBs:** Sugar Sweetened Beverages; **A:** Availability; **Av:** Variety as part of availability; **P:** Positioning; **OR:** Odds Ratio

**Table S6: Summary Table of Intervention Studies**

| **Author, Year, Country** | **Study Design** | **Setting** | **Participant Sample** | **Placement Strategy** | **Intervention Description** | **Outcome Measure** | **Key Findings** | **Effect  Summary*** | **Risk of Bias** |
| --- | --- | --- | --- | --- | --- | --- | --- | --- | --- |
| Adam et al.  (2017) ^S18^  Denmark | Quasi-experimental design  Matched control  BL: 5 weeks  Intervention period: 5 weeks | 10 supermarkets;  5 intervention  5 control  Stores matched on geographical location  No neighbourhood SES details | N/A | Position | **P:** Dairy items coded in relation to energy density (Red: high energy, Yellow: medium energy, Green: low density)  Dairy products then placed in differing shelf positions according to their energy code:  Green: favourable (middle and eye- level) position,  Yellow: Intermediate position,  Red: less favourable position  **Control:** Usual shelf allocation | Weekly sales of 80 dairy items coded as green, yellow or red according to energy density  Objective store sales data  [Results only present for combined green and red categories] | A significant effect of the shelf positioning intervention on calories sales of green dairy products was observed (β=0.09, SE=0.03, p<0.001)  No significant effect of the shelf positioning intervention on calories sales of red dairy products was observed (β=-0.04, SE=0.05, p>0.1) | 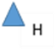  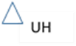 | High |
| Adjoian et al.  (2017) ^S19^  USA | Repeated cross-sectional measures  One control checkout per store  Measures recorded on 6 occasions over a 1-week period | 3 supermarkets  Deprived neighbourhood, New York | N/A | Position | **P:** Introduction of a healthy checkout containing products that met nutritional criteria (for kcal, fat, sat fat, trans fat, sodium, sugar, fibre):   - Nuts, seeds, dried F and/or trail mix - Granola bars - Fresh F/packaged F (apple sauce) - Bottled water or carbonated water - Chewing gum   Not all products on the checkout met the nutrition criteria  **Control:** 1 checkout in each store took part in the study but received no changes | % of customers, observed by researcher, purchasing healthy, unhealthy or neutral items from checkout area | More customers purchased healthy products when using the healthy checkout compared with those using the standard checkout (56.5% v 20.5%, p<=0.001)  Fewer customers using the healthy checkout purchased an unhealthy product compared with those using the standard checkout (45.7% v 74.4%, p=0.007) | 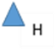  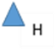 | High |
| Albert et al. (2017) ^S20^  USA | Repeated cross-sectional measures  Matched control  BL  FU: length of intervention period unclear, ranging from 1-2 years | 8 convenience stores;  3 intervention  5 control  (1 intervention store withdrew and became a control store  Stores matched on store characteristics (not described)  Majority Latino community, California | Intervention BL: n=264  Intervention FU: n=208  Control BL: n=286  Control FU: n= 199  Recruitment: In-store  Majority of respondents at both time points were:   - Female - Aged >43 years - Married or living with partner - Foreign born - Mexican heritage - High school education or lower | Position | **P:** F&V positioned at front of store and crisps/soft drink move to back of store  **Multi-component:** Store refurbishment, social marketing campaign  **Control:** No in-store intervention and no community work in surrounding neighbourhood. | Self-reported % of food dollars spent on F&V each week.  Self-report variety of F&V purchased during visit to store  Self-report total serving of F&V consumed per day. | No significant difference in % of food dollars spent on F&V was observed between the intervention and control groups for the study period (Intervention mean difference 1.7; Control mean difference 1.5, p>0.01)  A significant increase in mean number of F&V varieties purchased between BL and FU for intervention participants was observed (BL: 0.2 (SD 0.6), FU:0.5 (SD 1.1) p<0.001)  A significant difference in mean number of F&V varieties purchased was observed between the intervention and control group for the study period (Intervention mean difference 0.3; Control mean difference 0.0, p<0.001)  No significant difference in F&V intake/day was observed between intervention and control groups for the study period (Intervention mean difference 0.1; Control mean difference -0.3, p>0.01) | 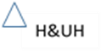    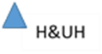  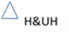    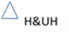 | High |
| Ayala et al. (2013) ^S21^  USA | Clustered randomised control trial  Delayed treatment control  BL  4 month FU | 4 convenience stores (Tiendas),  2 intervention  2 control  Majority Latino community, North Carolina | n=119  Recruitment: In-store (convenience sample)  BL: In-store  FU: Telephone  Age (mean): 32 years  Sex: 66% Female  Ethnicity: 85% Mexican  SES: Mean monthly income $US 1,500 | Availability  Position | **A&P**: New ready-to-eat F&V display at principal cash register  2-month duration  **Multi- component:** Staff training, food demonstrations, social marketing campaign  **Control:** Delayed treatment control | Self-report F&V consumption (portions/day)  Self- reported variety of F&V consumed  (Validated National Cancer Institute F&V All- Day Screener) | Increase in F&V consumption from 2.04 (1.19) to 2.88 (2.01) in the intervention group compared to 2.61 (1.53) to 2.84 (2.35) in the control group (p=0.06)  Variety of F consumed in the past month (out of 32) changed from 20.5 (5.75) to 20.93 (5.74) in the intervention group and 20.12 (5.49) to 19.13 (6.16) in the control (p>0.1)  Variety of V consumed in the past month (out of 43) changed from 25.93 (5.90) to 26.86 (7.77) in the intervention group and 26.42 (6.24) to 25.53 (7.54) in the control (p>0.1) | 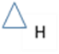  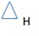    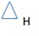 | High |
| Dannefer et al. (2012) ^S22^  USA | Repeated cross-sectional measures  No control group  BL  6-7 month FU | 8 convenience stores (Bodegas)  Deprived neighbourhoods, New York | BL: n= 294  FU: n=323  Recruitment: In-store (convenience sample)  BL: Exit interviews  FU: Exit Interviews  No demographic characteristics provided | Availability  Position | **A:** Increased variety of fresh F&V.  Increased availability of low salt canned V, canned F in F juice, whole-grain bread and 1% milk  **P:** Water displayed at eye-level  **Multi-component:**  Staff training, social marketing campaign | Self-report food and beverage purchases on store visit | Among 124 people at BL and 153 at FU who purchased a beverage, the percentage purchasing at least one bottle of water increased from 6% (n=8) to 12% (n=18)  Among the 111 customers at baseline and post intervention who purchased promoted healthier options, the percentage purchasing healthier options increased from 5% (n=6) before the intervention to 16% (n=18) after the intervention | 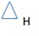    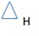 | High |
| De Wijk et al.  (2016) ^S23^  Netherlands | Alternating treatment time series  No control group  Intervention period: 12 weeks of 2 alternating treatments | 2 supermarkets in Veenendaal  No neighbourhood SES details | N/A | Position | 2 alternating treatments for the positioning of 5 types of bread (whole grain, dark wheat, wheat, light wheat, white) conducted in 2 periods  **P_1_:** Healthy bread first.  7- week period  Whole grain bread placed at entrance to aisle followed by dark wheat, wheat, light wheat and white)    **P_2_:** Healthy bread last.  5-week period  White bread placed at entrance to aisle followed by light wheat, wheat, dark wheat and whole grain) | Ratio:  Mean bread sales per week, for each bread type, for supermarket A: mean bread sales per week, for each bread type, for supermarket B  Objective store sales data | No significant effect of the manipulation on sales of the different bread types (F=1.90, p=0.174 for time period and F=1.95, p=0.115 for the interaction between type of bread and time period) | 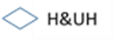 | High |
| Ejlerskov et al. (2018a1)^S24^  UK | Time series using repeated cross-sectional data  Matched control  Natural experiment evaluating supermarket chain-led checkout food policies  BL: 13 4-week periods  FU: 13 4-week periods | 9 UK supermarket chains;  6 intervention  3 control  Purchasing data representative of UK grocery market sales | n≈30,000 households  Purchasing of take-home food weighted and uplifted to represent n=27,385,050 households, aggregated into 4-weekly periods, to provide supermarket chain level data | Position | **P:** Voluntary supermarket chain-led checkout food policy limiting the positioning of unhealthy food items at checkouts  Policy presence identified through supermarket annual reports, webpages and press releases- intervention chains included those with i) clear and consistent (n=3) and ii) vague and inconsistent (n=3) policies  **Control:** Supermarket chains with no checkout food policy | Supermarket chain purchases per 4-week block of single-serve checkout foods (confectionery, chocolate and crisps collated)  Data from Kantar Wordpanel ‘take-home’ dataset | Compared to the counterfactual, implementation of supermarket chain-led checkout food policies was associated with statistically significant decreases in the purchasing of common checkout foods at:   - 4 weeks following the policy implementation (-157.7; CI95% -242.8, -72.7) - 12 months following the policy implementation (-185.1; CI95% -248.5, -121.7) | 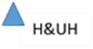  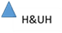 | High |
| Ejlerskov et al. (2018a2)^S24^  UK | Natural experiment evaluating supermarket chain-led checkout food policies  Matched control  Data collected annually from 2016-2017 | UK supermarket chains;  3 intervention  3 control | n≈7,500  Data weighted and uplifted to represent UK population n= 50, 398,000 individuals aged 13-79 years | Position | **P:** Voluntary supermarket chain-led checkout food policy limiting the positioning of unhealthy food items at checkouts  Policy types classified through supermarket annual reports, webpages and press releases- these analyses include i) clear and consistent (n=3) and ii) no checkout policy (n=3) | Annual household purchases of single-serve checkout foods (confectionery, chocolate and crisps collated) per market share  Data from Kantar Worldpanel ‘out-of-home’ dataset | When compared to supermarket chains with no checkout food policy, those with clear and consistent policies had significantly fewer annual unit purchases of common checkout foods per market share (β -25000; CI95% -37100, -12900) | 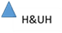 |  |
| Ejlerskov et al. (2018b) ^S25^  UK | Repeated cross-sectional data analysis  Natural experiment evaluating supermarket chain-led checkout food policies  Matched control  Data collected annually from 2013-2017 | UK supermarket chains;  6 intervention  4 control | n≈30,000 households  Data weighted and uplifted to represent all UK households n=27,385,050 households  Range of demographics across all supermarket chains:  Age (mean): 49.7- 58.6 years  Social class (means): 3.25-3.98 [5= most affluent] | Position | **P:** Voluntary supermarket chain-led checkout food policy limiting the positioning of unhealthy food items at checkouts  Policy types classified through supermarket annual reports, webpages and press releases- these analyses include i) clear and consistent (n=3) and ii) no checkout policy (n=4) | Annual household purchases of single-serve checkout foods (confectionery, chocolate and crisps collated) per market share  Annual purchases grouped according to social class (occupation of main household shopper)  Data from Kantar Worldpanel ‘take-home’ dataset | Significantly fewer purchases of common checkout foods per household per market share were made from supermarket chains with clear and consistent checkout food policies, when compared to purchases from supermarket chains with no checkout food policy (ratio 0.86; CI95% 0.78, 0.96)  A significant interaction between social class and checkout food policy was observed (p=0.02). When compared to all households, households in the two most affluent and the least affluent social class made fewer purchases of common checkout foods from supermarkets with a clear and consistent policy   - AB (lowest): (RGM^+^ 0.79, CI95% 0.65, 0.96) - C1: (RGM 0.74, CI95% 0.61, 0.91) - C2: (RGM 1.05, CI95% 0.86, 1.29) - D: (RGM 0.96, CI95% 0.79, 1.18) - E (Highest): (RGM 0.79, CI95% 0.65, 0.97) | 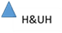  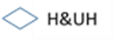 | High |
| Foster et al.  (2014) ^S26^  USA | Cluster randomised control trial  Matched control  BL: 3 month  FU: 6 month | 8 supermarkets from two large chains;  4 intervention  4 control  Stores matched on:   - Supermarket chain - Store size - Subsidised sales   Deprived neighbourhoods, Philadelphia and Wilmington | N/A | Availability  Position | **A:** Reduced shelf space of whole milk and full calorie soft drinks by 30%  **A:** Increased shelf space of reduced fat milk (2%, 1%, skim), lower calorie frozen meals, diet soft drinks and water  **P:** Reduced fat milk, low calorie frozen meals, diet soft drinks and water placed in more prominent shelf position  **Multi-component:** Signage, food demonstrations  **Control:** No intervention received | Difference between intervention and control stores in mean change of weekly sales from BL to FU for targeted products  Objective store sales data | Compared to control stores, intervention stores showed significantly greater mean change in sales of:  Skim milk (oz) (Mean change 1509.1 (SE 1079.9), p<0.01)  1% milk (oz) (Mean change 3383.2 (SE 1403.8), p<0.01)  Lower calorie frozen chicken nuggets (units) (Mean change 20.5 (SE 10.4), p<0.01)  Lower calorie frozen turkey dinner (units)  (Mean change 10.8 (SE 6.2), p<0.05)  Water in-aisle (oz) (Mean change 1690 (SE 6649.8), p<0.05)  Water checkout (units) (Mean change 18.5 (SE 6.0), p<0.01)  No significant differences in mean change of sales between intervention and control stores for other items:  Whole milk (oz) (Mean change -3910.6 (SE 4942.7), p=0.67)  2% milk (oz) (Mean change -2417.9 (SE 3410.9), p=0.55)  Lower calorie frozen steak meal (units) (Mean change 10.5 (SE 12.1), p=0.65)  Pepsi in-aisle (oz) (Mean change -2706.5 (SE 6250.6), p=0.88)  Diet Pepsi in-aisle (oz) (Mean change -507 (SE 970.5), p=0.29)  Regular checkout beverages (units) (Mean change -13.5 (SE 9.3), p=0.62)  Low Calorie checkout beverages (units) (Mean change 1.5 (SE 4.4), p=0.22) | 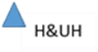 | Mod |
| Gittelsohn et al.  (2010) ^S27^  USA | Quasi-experimental design    Unmatched control  BL  FU: 19 months | 13 convenience stores and 3 supermarkets;  Intervention: 7 convenience stores (Korean) and 2 supermarkets in East Baltimore  Control: 6 convenience stores (Korean) and 1 supermarket in West Baltimore  Deprived neighbourhoods with majority African American community, Baltimore | n= 83  Recruitment: In-store and community action centres (convenience sample)  FU: In person interview  No demographic characteristics provided | Availability | Intervention was delivered in 5 phases, each lasting 2 months:  **A:** Increased availability of:   1. Low sugar cereal, high-fibre cereals and low-fat milk 2. Cooking oil spray 3. Fresh F and low fat snacks 4. Wholegrain bread and low fat mayonnaise 5. Water and diet carbonated drinks   **Multi-component:** Signage, food demonstrations, staff training, financial incentives for store owners  **Control:** No intervention received | Self-reported ‘healthy food getting’ assessing consumption of 26-foods in previous 30-days from all food sources including shops, church, food stamps, friends and family. | ‘Healthy food getting’ scores (consumption), from BL to FU, showed a non-significant reduction in the intervention group compared to the control group (β= -0.09, p=0.4) |  | High |
| Holmes et al.  (2012) ^S28^  USA | Time series  No control group  BL: 5 weeks  FU:12 weeks | 1 supermarket  Majority white neighbourhood, Roanoke | N/A | Availability  Position | **A&P:** New child targeted display in a prominent location promoting 32 food items including F&V and healthier child friendly diary, meat and cereal products  **Multi-component**: Food demonstrations | Change in mean weekly sales of targeted items as proportion of total sales  Objective store sales data  (We only present results for F&V, whole-grain products and sunflower seeds total n=15) | 4 items showed significant increases in sales during the intervention period (p<0.05) (whole wheat bagels, sunflower seeds, bananas and radishes)  6 other items showed an increase in sales but were not statistically significant (yellow peppers, carrots, lemon, apple, kiwi, non-fat milk)  2 items showed significant decreases in sales during the intervention period (p<0.05) (broccoli and tomatoes)  4 items showed decreased sale but were not statistically significant (pineapple, mango, orange, whole-wheat pita)  Add detail about effect sizes/ change in proportion  (Due to the difficulty in classifying some food items as healthy/ unhealthy without nutritional information, in some cases we only report the foods that can be easily classified in accordance to the dietary guidelines e.g. F&V) |  | High |
| Jilcott Pitts et al. (2018) ^S29^  USA | Repeated cross-sectional study  Matched controls    Natural experiment evaluating North Carolina Healthy Food Small Retailer Program  BL  FU: 6 months | 8 convenience stores; 4 intervention,  4 control  Stores matched on:   - Store type - Store size - Food desert type - % SNAP - % African American   8 census tract areas   - 15-53% SNAP - 23-88% African American | BL: n= 279  FU: n= 223  Recruitment: In-store (convenience sample)  Demographics across data collection points:  Age (mean): 42.5 - 44.9 years  Sex: 30.3-44.2% Female  Ethnicity: 39.5-87.2% Black | Availability | Intervention included a $25,000 grant to increase the availability of healthy foods (F&V, low-fat milk, whole grain products) in stores  Intervention was unique to each store  **A:** Assessed by Healthy Food Supply (HFS) score. On average, intervention stores increased by 3.13 points and control stores decreased by -0.44 points from pre study to FU.  [HFS score (possible range 0-31) summarises availability, quality, variety and price of food in store. Higher scores represent healthier stores.]  **Multi-component:** Promotional events, purchasing of display equipment  **Control:** Did not receive $25,000 grant to make adaptations to store | Store-level healthfulness of sales from bag check data using HEI-2010  Participant self-reported consumption of:   1. F&V 2. SSBs   Participant skin carotenoid status measured by pressure mediated reflection spectroscopy  Participant BMI (km/m^2^) using self-reported height and weight | A non-significant difference between groups in mean change of store-level HEI scores was observed, with scores decreasing in both intervention and control groups (Intervention store mean difference = -1.19; control stores mean difference = -0.08; p= 0.83)  A non-significant difference between intervention and control groups in mean daily F&V intake was observed with consumption increasing among intervention participants (β= 0.68, SE=0.64; p=0.29)  A non-significant difference between intervention and control groups in mean SSB intake was observed with consumption decreasing among intervention participants (β= -0.25, SE=0.37; p=0.50)  A non-significant difference between intervention and control groups in mean skin carotenoids was observed, indicating consumption decreased among intervention participants  (β= -7.92, SE=14.77; p=0.59)  A non-significant difference between intervention and control groups in mean BMI was observed, indicating an increased BMI among intervention participants (β= 0.67, SE=1.27; p=0.60) |  | High |
| Lawman et al.  (2015) ^S30^  USA | Repeated cross-sectional measures  No control group  BL  FU: 12 months | BL: n= 173  FU: n= 113  Convenience stores  Deprived neighbourhoods, Philadelphia | BL: n= 8671  FU: n= 5949  Recruitment: In-store (convenience sample)  Sex: 41% Female (BL & FU) | Availability | **A:** Introduction of 4 new healthy foods (2 new products from 2 different groups: fresh F&V, canned / dried F&V, low fat diary, lean meats, whole grains)  **Multi-component:** social marketing campaign, staff training | Change in energy (kcal), fat (g), protein (g), carbohydrates (g), sugar (g), fibre (g) and sodium (mg) in foods purchased at BL and FU  Researcher conducted CPA when exiting store  [Results only present for fibre, sugar and sodium due to clear relationship with health] | A non- significant reduction in sugars (g) was observed between intervention BL and FU: (Change -0.37; SE 2.77; p=0.85)  A non-significant increase in dietary fibre (g) was observed between intervention BL and FU: (Change 0.08; SE 0.14; p=0.56)  A non-significant increase in sodium (mg) was observed between intervention BL and FU: (Change 31.49; SE 89.55; p= 0.72) |  | High |
| Sigurdsson et al. (2009) ^S31^  Iceland | Alternating treatment time series  No control group  BL: 44-52 days  Intervention period: 72 days of 4 interventions | 2 discount supermarkets in Reykjavik  No neighbourhood SES details | N/A | Availability  Position | **A&P:** Introduction of a large display at the front of the store with 112 facings of the target potato crisp brand  **P:** Alternating placement of target brand of potato crisps between low (24cm), middle (123cm) and high shelves (173cm) | Sales of target potato chip brand as a percentage of total potato chip sales (24 brands including target brand)  Objective store sales data | Mean relative sales of the target brand were highest when positioned on the middle shelf compared to positioning on low and high shelf in both stores (Store A: middle 7.5%, range 6.6%-8.6%; low 4%, range 2.9%- 5.8%; high 3.3%, range 2.7%-3.6%. Store B: middle 5.7%, range 5%-6.2%; low 4.4%, range 4.1%- 5%; high 4.4%, range 4.1%-4.7%.)  Mean relative sales of target crisp brand during presence of extra display at the front of store, appeared to be higher than BL in both stores (store A:12.6%, range 8.3%-15.7%; store B: 9%, range 3% -12.3%) |  | High |
| Sigurdsson et al. (2011) ^S32^  Norway | Alternating treatment time series  No control group  Intervention period: 56 days of 3 interventions and baseline | 1 small supermarket  1 discount supermarket  No neighbourhood SES details | N/A | Position | 3 interventions alternating with baseline condition for periods of 4 days  **P_1_:** Bananas in aisle and at checkout (alongside confectionery)  **P_2_:** Bananas in aisle and on confectionery shelf  **P_3_:** Bananas in aisle, at checkout and on confectionery shelf (and shelf prompts)  **Multi-component:** Signage | Mean proportion of banana sales compared to total F sales (including bananas) for each condition of the study  Objective store sales data | Mean sales of bananas increased from BL (small: 26.38%, range: 22.35- 30.23%; discount: 16.94%, range: 15.56- 17.79%) for the checkout position (small: 27.58%, range 27.17-28.08%; discount: 18.24%, range15.96-20.54%)  Mean sales of bananas for the confectionery shelf condition were inconsistent  Mean sales of bananas increased from BL for checkout, confectionery shelf and shelf prompt condition (small: 28.46%, range 25.34-31.57%; discount: 21.45%, range 20.26-22.64%) |  | High |
| Sigurdsson et al. (2014) ^S33^  Norway | Alternating treatment time series  No control group  BL:12-16 days  Intervention period: 12-40 days alternating 2 interventions and baseline.  FU:12 days | 1 small supermarket  1 discount supermarket  No neighbourhood SES details | N/A | Position | 2 interventions alternating with baseline condition for periods of 4 days  **P_1_:** Dried fish and dried F & nut mix located at checkouts with confectionary and chewing gum next to checkout  **P_2_ (Multi-component):** P_1_ plus signage  BL: Confectionary and chewing gum positioned at checkout. Dried fish and dried F & nut mix positioned in standard location | Total unit of sales of targeted products in 4-day period  Objective store sales data | Sales of dried fish and dried F & nut mix increased relative to BL for both the store position only and the store position + shelf prompt condition for both products (~15-200% no specific details given)  Sales of confectionary and chewing gum reduced slightly in one store during the intervention phase and remained lower than BL in the FU period |  | High |
| Song et al.  (2009) ^S34^  USA | Quasi-experimental design (feasibility study)  Unmatched control  BL  FU: 10 months | 13 convenience stores (Korean)  Intervention: 7 convenience stores in East Baltimore  6 control in West Baltimore  Deprived neighbourhoods with majority African American community, Baltimore | N/A | Availability | Intervention was delivered in 5 phases, each lasting 2 months:  **A:** Increase availability of:   1. Low sugar cereal, high-fibre cereals and low-fat milk 2. Cooking oil spray 3. Fresh F and low fat snacks 4. Wholegrain bread and low fat mayonnaise 5. Water and diet carbonated drinks   **Multi-component:** Signage, food demonstrations, staff training, financial incentives for store owners  **Control:** No intervention received  [Intervention details taken from Gittelsohn et al, 2010 due to lack of detail in manuscript] | Sales of 10 promoted healthy foods reported for the previous 7 days  Binary measures (0= no units sold/ week, 1= 1 or more units sold/ week) for each product self-reported by store managers | Compared to control stores, a positive intervention association was observed for:  Cooking spray (Intervention mean 0.3(SD 0.5); Control mean -0.5 (SD 0.6); p=0.05)    Low sugar cereal (Intervention mean 3.9 (SD8.8; Control mean -1.9 (SD1.4); p=0.13)  Baked/low-fat crisps (Intervention mean 1.1 (SD 2.0); Control mean 0 (SD 0); p=0.13)  Low-salt crackers (Intervention mean 0.3(SD 0.8); Control mean -0.4 (SD 0.7); p=0.13)  Whole wheat bread (Intervention mean 0.4 (SD 6.2); Control mean -1.9 (SD 8.3); p=0.71)  100% F juice (Intervention mean 4.9 (SD 39.4); Control mean -10.6 (SD 59.8); P=0.63)  Compared to control stores, a positive intervention association was observed for:  High fibre cereal (Intervention mean -0.8 (SD 2.1); Control mean -2.8 (SD 2.6); p=0.20)  Low fat milk (Intervention mean -0.4 (SD 1.3); Control mean -1.9 (SD2.7); p=0.23)  Diet beverages (Intervention mean -10.0 (SD 39.2); Control mean 10.5 (SD 29.5); p=0.58)  Water (Intervention mean -15.8(SD 91.9); Control mean -2.2 (SD 56.5); P=0.83) |  | High |
| Thorndike et al. (2017) ^S35^  USA | Randomised control trial (pilot study)  Matched controls  BL: 11 months  Intervention period: 5 months | 6 convenience stores accepting both WIC and SNAP vouchers;  3 intervention  3 control  Stores matched on monthly WIC sales  Deprived neighbourhoods with majority Hispanic community, Chelsea, Massachusetts | n=575 over 2 cross-sectional survey  Recruitment: In-store  n=295 intervention  Sex: 53% Female  Age: 55% 18-39years  37% 40-59 years  8% >=60years  Ethnicity: 82% Hispanic/ Latino  n=280 control  Sex: 57% Female  Age: 56% 18-39years  38% 40-59 years  6% >=60years  Ethnicity: 92% Latino/ Hispanic | Position | **P:** Positioning fresh F&V at the front of the store (replacing baked goods in one store and crisp display in another)  **Multi-component:** Staff-training, store refurbishment | WIC F&V voucher sales ($US) per store per month. Data received from WIC state office  Self-reported purchases of F&V by customers exiting the store | During the intervention period, F&V WIC voucher sales increased in intervention stores by $40/month and decreased in control stores by $23/month. (Difference in trend: $63/month (CI $4-121), p=0.036)  No significant difference in % of customers purchasing fresh F&V for intervention and control store customers between BL and intervention periods (Difference: intervention 1; control -4%, p=0.29) |  | Mod |
| Toft et al.  (2017) ^S36^  Denmark | Quasi- experimental design  Unmatched control  BL: 1 month  Intervention period: 3 months  Post intervention: 1 month | 3 supermarkets;  1 intervention  2 control  No neighbourhood SES details | N/A | Availability  Position | **A:** Increased stocking of F&V (mainly fresh)  **P:** F&V placed in produce bins in high traffic areas such as at the entrance, end –caps and near the checkout. F&V replaced non-food items snacks and confectionary in these areas  **Control:** No clear description | Weekly store sales data for all F&V, confectionery, sugary beverages, cakes, fish and wholegrain products.  Index created for each store [number sold products in intervention year/ number products sold in previous year) x 100]  Objective store sales data. | A non-significant increase in total F&V sales was observed for the intervention (estimate 0.02 (SD 0.06), p=0.72)  A non-significant increase in fresh V sales was observed for the intervention (estimate 0.04 (SD 0.06) p=0.496)  A non-significant decrease in fresh F sales was observed for the intervention (estimate -0.0016 (SD 0.09) p=0.100)  A non-significant increase in organic fresh F&V sales was observed for the intervention (estimate 0.0231 (SD 0.06) p=0.680)  A significant decrease of 14.3% in cake sales was observed for the intervention (estimate -0.14, p=0.046)  A non-significant decrease of 0.07% in confectionery sales was observed for the intervention (estimate -0.07, p=0.09) |  | High |
| Wensel et al.  (2019) ^S37^  USA | Randomised controlled pilot trial  Unmatched control  BL: 2 months  Post intervention FU: 1 month | 4 convenience stores;  2 intervention (position only)  2 control  Deprived neighbourhoods with majority African American community, Baltimore | N/A | Position | **P:** WIC eligible food products moved to the front of the store and/or to eye level.  Intervention was implemented for 1 month  **Control:** No treatment | Change from BL to FU in total sales of WIC eligible food products at store level in past 7 days  Change from BL to FU in total sales of WIC eligible food products to WIC consumers in past 30 days  Sales data reported by the store owner | Product positioning only strategies showed a non-significant difference between intervention and control groups (p>0.05), with sales decreasing among intervention stores for:   - Total store sales β -35.5 (SE 54.1) - Total sales to WIC customers β -76.5 (SE 39.7) |  | High |
| Winkler et al.  (2016) ^S38^  Denmark | Repeated cross-sectional measures  No control stores  BL: 4 weeks  Intervention period: 4 weeks  Post intervention:7 weeks^1^ | 4 supermarkets  in Bornholm  No neighbourhood SES details | N/A | Position | **P:** Healthy snack products displayed at one checkout above the conveyor belt in each store, replacing sugar confectionery. Most commonly healthy snack products included:   1. Fresh F 2. Dried F 3. Dried F bars 4. Carrot snack packs | Weekly store sales data for all sugar confectionary and most common intervention items (fresh F, dried F, dried F bars, carrot snack packs)  Objective store sales data | A non- significant reduction in sugar confectionary sales was observed for the intervention period when compared to the pre and post intervention periods (Pre: 0.93 (CI 0.80-1.06); Post: 0.96 (0.84-1.11)  A non- significant reduction in fresh F sales was observed for the intervention period when compared to the pre and post intervention periods (Pre: 0.94 (CI 0.80-1.11); Post: 0.92 (0.78-1.09)  A non- significant reduction in dried F sales was observed for the intervention period when compared to the pre and post intervention periods (Pre: 0.80 (CI 0.58-1.11); Post: 0.86 (0.62-1.19)  A non- significant increase in F bar sales was observed for the intervention period when compared to the pre and post intervention periods (Pre: 1.31 (CI 0.78-2.20); Post: 1.37 (0.82-2.30)  A non- significant increase in carrot snack pack sales was observed for the intervention period when compared to the pre and post intervention periods (Pre: 1.01 (CI 0.73-1.39); Post: 1.13 (0.82-1.56) |  | High |

**Effect summary:** Direction of result and significance level **(** Positive result p<0.05;Positive result p>0.05;Negative result p<0.05**;** Negative result p>0.05),and health rating of intervention (H: Healthy; UH: Unhealthy; H&UH: Healthy and Unhealthy items were considered as the intervention);
**BL:** Baseline**; FU:** Follow- Up**; SES:** Socio- Economic Status**; F&V:** Fruit and vegetables**; F:** Fruit; **V:** Vegetables; **CPA:** Customer Purchase Assessment; **WIC**: Women, Infants, Children; **SNAP**: Supplemental Nutrition Assistance Program; **RGM:** Ratio of Geometric Mean

**Supplementary References**

1. Bodor JN, Rose D, Farley TA, Swalm C, Scott SK. Neighbourhood fruit and vegetable availability and consumption: the role of small food stores in an urban environment. *Public health nutrition.* 2007;11(4):413-420.
2. Caldwell EM, Miller Kobayashi M, DuBow WM, Wytinck SM. Perceived access to fruits and vegetables associated with increased consumption. *Public health nutrition.* 2009;12(10):1743-1750.
3. Caspi CE, Lenk K, Pelletier JE, et al. Association between store food environment and customer purchases in small grocery stores, gas-marts, pharmacies and dollar stores. *The international journal of behavioral nutrition and physical activity.* 2017;14(1):76.
4. Cohen DA, Collins R, Hunter G, Ghosh-Dastidar B, Dubowitz T. Store Impulse Marketing Strategies and Body Mass Index. *American journal of public health.* 2015;105(7):1446-1452.
5. Franco M, Diez-Roux AV, Nettleton JA, et al. Availability of healthy foods and dietary patterns: the Multi-Ethnic Study of Atherosclerosis. *The American journal of clinical nutrition.* 2009;89(3):897-904.
6. Gustafson A, Christian JW, Lewis S, Moore K, Jilcott S. Food venue choice, consumer food environment, but not food venue availability within daily travel patterns are associated with dietary intake among adults, Lexington Kentucky. *Nutrition journal.* 2013;12:17.
7. Gustafson AA, Sharkey J, Samuel-Hodge CD, et al. Perceived and objective measures of the food store environment and the association with weight and diet among low-income women in North Carolina. *Public health nutrition.* 2011;14(6):1032-1038.
8. Jani R, Rush E, Crook N, Simmons D. Availability and price of healthier food choices and association with obesity prevalence in New Zealand Māori. *Asia Pacific journal of clinical nutrition.* 2018;27(6):1357-1365.
9. Jilcott Pitts SB, Wu Q, Truesdale KP, et al. Baseline assessment of a healthy corner store initiative: Associations between food store environments, shopping patterns, customer purchases, and dietary intake in eastern North Carolina. *International Journal of Environmental Research and Public Health.* 2017;14(10).
10. Kerr J, Sallis JF, Bromby E, Glanz K. Assessing reliability and validity of the GroPromo audit tool for evaluation of grocery store marketing and promotional environments. *Journal of nutrition education and behavior.* 2012;44(6):597-603.
11. Martin KS, Havens E, Boyle KE, et al. If you stock it, will they buy it? Healthy food availability and customer purchasing behaviour within corner stores in Hartford, CT, USA. *Public health nutrition.* 2012;15(10):1973-1978.
12. Nakamura R, Pechey R, Suhrcke M, Jebb SA, Marteau TM. Sales impact of displaying alcoholic and non-alcoholic beverages in end-of-aisle locations: an observational study. *Social science & medicine.* 2014; 108:68-73.
13. Rose D, Hutchinson PL, Bodor JN, et al. Neighborhood food environments and Body Mass Index: the importance of in-store contents. *American journal of preventive medicine.* 2009;37(3):214-219.
14. Ruff RR, Akhund A, Adjoian T. Small Convenience Stores and the Local Food Environment: An Analysis of Resident Shopping Behavior Using Multilevel Modeling. *American journal of health promotion.* 2016;30(3):172-180.
15. Sanchez-Flack J, Pickrel JL, Belch G, et al. Examination of the Relationship between In-Store Environmental Factors and Fruit and Vegetable Purchasing among Hispanics. *Int J Environ Res Public Health.* 2017;14(11).
16. Thornton LE, Crawford DA, Ball K. Neighbourhood-socioeconomic variation in women's diet: the role of nutrition environments. *European journal of clinical nutrition.* 2010;64(12):1423-1432.
17. Thornton LE, Cameron AJ, Crawford DA, McNaughton SA, Ball K. Is greater variety of chocolates and confectionery in supermarkets associated with more consumption? *Australian and New Zealand journal of public health.* 2011;35(3):292-293.
18. Adam A, Jensen JD, Sommer I, Hansen GL. Does shelf space management intervention have an effect on calorie turnover at supermarkets? *Journal of Retailing and Consumer Services.* 2017; 34:311-318.
19. Adjoian T, Dannefer R, Willingham C, Brathwaite C, Franklin S. Healthy Checkout Lines: A Study in Urban Supermarkets. *Journal of Nutrition Education and Behavior.* 2017;49(8):615-622.e611.
20. Albert SL, Langellier BA, Sharif MZ, et al. A corner store intervention to improve access to fruits and vegetables in two Latino communities. *Public Health Nutrition.* 2017;20(12):2249-2259.
21. Ayala GX, Baquero B, Laraia BA, Ji M, Linnan L. Efficacy of a store-based environmental change intervention compared with a delayed treatment control condition on store customers' intake of fruits and vegetables. *Public health nutrition.* 2013;16(11):1953-1960.
22. Dannefer R, Williams DA, Baronberg S, Silver L. Healthy bodegas: Increasing and promoting healthy foods at corner stores in New York City. *American Journal of Public Health.* 2012;102:e27-e31.
23. de Wijk RA, Maaskant AJ, Polet IA, Holthuysen NTE, van Kleef E, Vingerhoeds MH. An In-Store Experiment on the Effect of Accessibility on Sales of Wholegrain and White Bread in Supermarkets. *PloS one.* 2016;11(3):e0151915.
24. Ejlerskov KT, Sharp SJ, Stead M, Adamson AJ, White M, Adams J. Supermarket policies on less-healthy food at checkouts: Natural experimental evaluation using interrupted time series analyses of purchases. *PLoS medicine.* 2018;15(12).
25. Ejlerskov K, Sharp SJ, Stead M, Adamson AJ, White M, Adams J. Socio-economic and age variations in response to supermarket-led checkout food policies: a repeated measures analysis. *International Journal of Behavioral Nutrition and Physical Activity.* 2018;15(1):125.
26. Foster GD, Karpyn A, Wojtanowski AC, et al. Placement and promotion strategies to increase sales of healthier products in supermarkets in low-income, ethnically diverse neighborhoods: a randomized controlled trial. *The American journal of clinical nutrition.* 2014;99(6):1359-1368.
27. Gittelsohn J, Song HJ, Suratkar S, et al. An urban food store intervention positively affects food-related psychosocial variables and food behaviors. *Health education & behavior : the official publication of the Society for Public Health Education.* 2010;37:390-402.
28. Holmes AS, Estabrooks PA, Davis GC, Serrano EL. Effect of a grocery store intervention on sales of nutritious foods to youth and their families. *Journal of the Academy of Nutrition and Dietetics.* 2012;112(6):897-901.
29. Jilcott Pitts SB, Wu Q, Truesdale KP, et al. One-Year Follow-Up Examination of the Impact of the North Carolina Healthy Food Small Retailer Program on Healthy Food Availability, Purchases, and Consumption. *International journal of environmental research and public health.* 2018;15(12):2681.
30. Lawman HG, Vander Veur S, Mallya G, et al. Changes in quantity, spending, and nutritional characteristics of adult, adolescent and child urban corner store purchases after an environmental intervention. *Preventive medicine.* 2015;74:81-85.
31. Sigurdsson V, Saevarsson H, Foxall G. Brand Placement and Consumer Choice: an in-store experiment. *Journal of applied behavior analysis.* 2009;42:741-745.
32. Sigurdsson V, Larsen NM, Gunnarsson D. An in-store experimental analysis of consumers' selection of fruits and vegetables. *The Service Industries Journal.* 2011;31(15):2587-2602.
33. Sigurdsson V, Larsen NM, Gunnarsson D. Healthy food products at the point of purchase: An in-store experimental analysis. *Journal of Applied Behavior Analysis.* 2014;47:151-154.
34. Song HJ, Gittelsohn J, Kim M, Suratkar S, Sharma S, Anliker J. A corner store intervention in a low-income urban community is associated with increased availability and sales of some healthy foods. *Public health nutrition.* 2009;12(11):2060-2067.
35. Thorndike AN, Bright OM, Dimond MA, Fishman R, Levy DE. Choice architecture to promote fruit and vegetable purchases by families participating in the Special Supplemental Program for Women, Infants, and Children (WIC): randomized corner store pilot study. *Public Health Nutr.* 2017;20(7):1297-1305.
36. Toft U, Winkler LL, Mikkelsen BE, Bloch P, Glumer C. Discounts on fruit and vegetables combined with a space management intervention increased sales in supermarkets. *European journal of clinical nutrition.* 2017;71(4):476-480.
37. Wensel CR, Trude ACB, Poirier L, et al. B’more healthy corner stores for moms and kids: Identifying optimal behavioral economic strategies to increase WIC redemptions in small Urban corner stores. *International Journal of Environmental Research and Public Health.* 2019;16(1).
38. Winkler LL, Christensen U, Glumer C, et al. Substituting sugar confectionery with fruit and healthy snacks at checkout - a win-win strategy for consumers and food stores? a study on consumer attitudes and sales effects of a healthy supermarket intervention. *BMC public health.* 2016;16(1):1184.
